# Novel Smartphone App and Supportive Accountability for the Treatment of Childhood Disruptive Behavior Problems: Protocol for a Randomized Controlled Trial

**DOI:** 10.2196/67051

**Published:** 2025-03-11

**Authors:** Oliver Lindhiem, Claire S Tomlinson, David J Kolko, Jennifer S Silk, Danella Hafeman, Meredith Wallace, I Made Agus Setiawan, Bambang Parmanto

**Affiliations:** 1 University of Pittsburgh Pittsburgh, PA United States

**Keywords:** mobile health, disruptive behaviors, parent management training, randomized controlled trial, externalizing behavior

## Abstract

**Background:**

Although evidence-based treatments have been developed for childhood behavior problems, many families encounter barriers to treatment access and completion (eg, local availability of services, transportation, cost, and perceived stigma). Smartphone apps offer a cost-efficient method to deliver content to families.

**Objective:**

The aim of this study is to evaluate the effectiveness of the UseIt! mobile health system as both stand-alone and coach-assisted interventions via a randomized controlled trial. The UseIt! System is designed to reduce disruptive behaviors in young children.

**Methods:**

A nationwide sample of parents of children aged 5 years to 8 years with disruptive behaviors (N=324 dyads) are randomly assigned to the stand-alone app (UseIt!; n=108), the coach-assisted app (UseIt! plus supportive accountability; n=108), or the control app (mindfulness app; n=108). The UseIt! App provides parents with tools and troubleshooting to address disruptive behaviors, along with a behavior diary to track behaviors and strategies over time. The coach-assisted condition includes a bachelor’s level paraprofessional who provides weekly phone calls to promote engagement with the app. The control condition is composed of a mindfulness app. The web-based, self-assessed outcome measures (post treatment and 6-month follow-up) include measures of app usage, parenting knowledge (eg, knowledge of parent management training and cognitive behavioral therapy skills), and strategies (use of evidence-based parenting strategies), symptom reduction (eg, behavior problems), and parent mental health (eg, anxiety, stress, and depression). We hypothesize that both intervention conditions will show greater parent knowledge and use of skills along with greater symptom reduction relative to the control condition. Further, we hypothesize that those assigned to the coach assisted condition will report greater knowledge, skill use, and symptom reduction than the stand-alone app. We will use intent-to-treat analyses to regress outcomes on study conditions to evaluate for differences across conditions.

**Results:**

Recruitment of study participants began in December of 2022 and is ongoing. We have recruited over half of our intended sample of 324 parent-child dyads (n=214) as of December 2024. These dyads have been randomly allocated to each of the intervention conditions, with 71 assigned to the coach-assisted condition, 72 assigned to the stand-alone app, and 71 assigned to the control app condition. Data collection is projected to be completed by late 2026.

**Conclusions:**

The current study aims to address a gap in the literature regarding the feasibility, effectiveness, and utility of a smartphone app that includes a coach-assisted arm. Digital therapeutics have the potential to enhance the reach and scalability of skills-based psychosocial interventions. Findings from this study will advance scientific knowledge and have implications for clinical practice.

**Trial Registration:**

ClinicalTrials.gov NCT05647772; https://clinicaltrials.gov/study/NCT05647772

**International Registered Report Identifier (IRRID):**

DERR1-10.2196/67051

## Introduction

### Background and Rationale

In the United States, approximately 1.5 million school-age children meet *DSM-5* (*Diagnostic and Statistical Manual of Mental Disorders* [Fifth Edition]) diagnostic criteria for a disruptive behavior disorder (DBD) [1]. DBDs, a category of disorders that broadly involves problems concerning the self-control of behaviors (eg, conduct disorder [CD] and oppositional defiant disorder [ODD]), account for more than half of all mental health referrals for children [2,3]. Longitudinal studies reveal that these problems in early childhood can be risk factors for persistent problems later in life, including substance use disorders and internalizing disorders, if left untreated [4]. DBDs are typically treated with psychosocial evidence-based therapies [5] that include parent management training (PMT) skills (eg, praise, rewards, consequences, and time-outs) and cognitive behavioral therapy (CBT) skills (eg, problem-solving and emotion labeling) [6]. Meta-analyses point to the substantial effectiveness of these interventions at reducing symptoms and maintaining treatment gains over time [7,8]. Despite the effectiveness of evidence-based treatments, many families do not have access to these services, and often stop attending or fail to practice new skills between sessions. Barriers to access include local availability of services, transportation, cost, and perceived stigma. Barriers to noncompletion include poor motivation and low engagement, along with competing demands for time, transportation problems, and copayment costs [9].

### The Promise of Digital Therapeutics

Recent advances in technology, in particular mobile health (mHealth) systems, have the potential to overcome these barriers and promote better data collection for researchers [10]. mHealth technologies, including smartphones, create an opportunity to develop personalized interventions that are delivered to families in their day-to-day settings [11]. A smartphone-based mHealth system has numerous potential advantages for improving access to, and engagement in, evidence-based treatments for childhood behavior problems. Such applications can deliver content to improve understanding of skills, provide opportunities for learning through skills practice, and give feedback to families regarding areas for improvement [12,13]. In a recent meta-analysis, of 25 clinical trials, mobile technology use was associated with superior treatment outcome across all study designs and types of control conditions (effect size=.34) [14]. mHealth technologies can also be used to collect data on between-session treatment adherence and skills practice. Evaluating the extent to which patients and families are using the skills they are learning outside of treatment sessions is especially important for psychosocial treatments for disruptive behavior problems which are overwhelmingly skills-based. Remote digital assessments have many advantages over traditional retrospective self-reports including reduced recall bias, the ability to obtain a more representative sample of behavior across situations and contexts, and the ability to track changes in behavior [15-17].

Rigorous evaluations of mHealth interventions targeting child behavioral concerns are in their nascent stage. A 2024 systematic review of mHealth interventions targeting behavioral problems in youth indicated a wide range of effect sizes, from small to large, across the 11 studies reviewed. Most sample sizes were small (ie, less than 100), and the few larger studies did not focus on clinical outcomes (eg, satisfaction, acceptability, and app usage, without behavioral outcomes) [18]. Other recent studies reflect similar findings (ie, improvements in child and parental symptoms, with a wide range of effect sizes), but have similar limitations (ie, small sample sizes, outcome measures focused on satisfaction or acceptability of apps rather than empirically based measures of symptom improvement) [19-22].

### The Role of the Coach in mHealth

There is growing evidence in the field of digital therapeutics that some degree of human interaction is important to sustain app usage and achieve meaningful outcomes [23-25]. Although various models have been proposed, the “Coaching” model affords many of the benefits of human interaction (eg, support and accountability) at a level of service that remains highly scalable [23]. Mohr’s Supportive Accountability (SA) Model, which is flexible and can be tailored to clinical conditions and service users, details that the intervention is supported by a coach who provides a social presence and accountability to boost motivation and engagement. Effectiveness studies have shown that various paraprofessionals can successfully be trained as coaches for a wide range of interventions and clinical populations [23].

### Previous Pilot Trials of the UseIt! mHealth System

The UseIt! app combines aspects of PMT and CBT to provide parents with evidence-based skills to decrease disruptive behaviors in their children. To date, we have completed 2 pilot randomized controlled trials (RCTs) to test preliminary target engagement and effectiveness of the UseIt! mHealth system. The first trial tested the UseIt! mHealth system as a stand-alone intervention (N=34). Parent-child dyads enrolled in the study and were randomly assigned to either the UseIt! mHealth app condition (n=17) or to a waitlist condition (n=17). Overall, results supported the feasibility of the intervention, but attrition was high in the waitlist control group. This informed our decision to have an active control group rather than a waitlist control group in the current study. The second pilot trial tested the UseIt! mHealth system as an adjunct to therapy in community settings (N=39 parent-child dyads). Though treatment targets moved in the expected direction, high clinician turnover in community settings limited the sustainability and scalability of this approach. This informed our decision to include a bachelor’s level paraprofessional “coach” rather than a clinician in this study.

### Study Aims and Hypotheses

#### Primary Aim

We aim to evaluate the effectiveness of the UseIt! mHealth system as both a stand-alone (n=108) and coach-assisted (n=108) intervention compared with a control app condition (n=108). We expect that (1) the 2 UseIt! intervention conditions will score higher on parenting knowledge (primary outcome) and show greater posttreatment reductions in disruptive behavior symptoms (secondary outcome) compared with the control condition, and (2) the coach-assisted UseIt! condition will score higher on parenting knowledge and show greater posttreatment reductions compared to the stand-alone UseIt! condition.

#### Secondary Aims

We will also test mechanisms of therapeutic change. In particular, we will test whether gains in knowledge of parenting skills are associated with reductions in disruptive behavior symptoms. Finally, we aim to evaluate the effectiveness of the components of UseIt! mHealth system. We will compare app usage across the stand-alone and coach-assisted conditions and test whether the app usage indices are associated with target engagement, knowledge of parenting skills, and symptom reduction at post-treatment. We expect that families who use the app more often will have higher skill acquisition and usage scores at post treatment (“dose” effects) though we do not have specific hypotheses regarding individual app features.

## Methods

### The UseIt! Smartphone App

**Figure 1:**
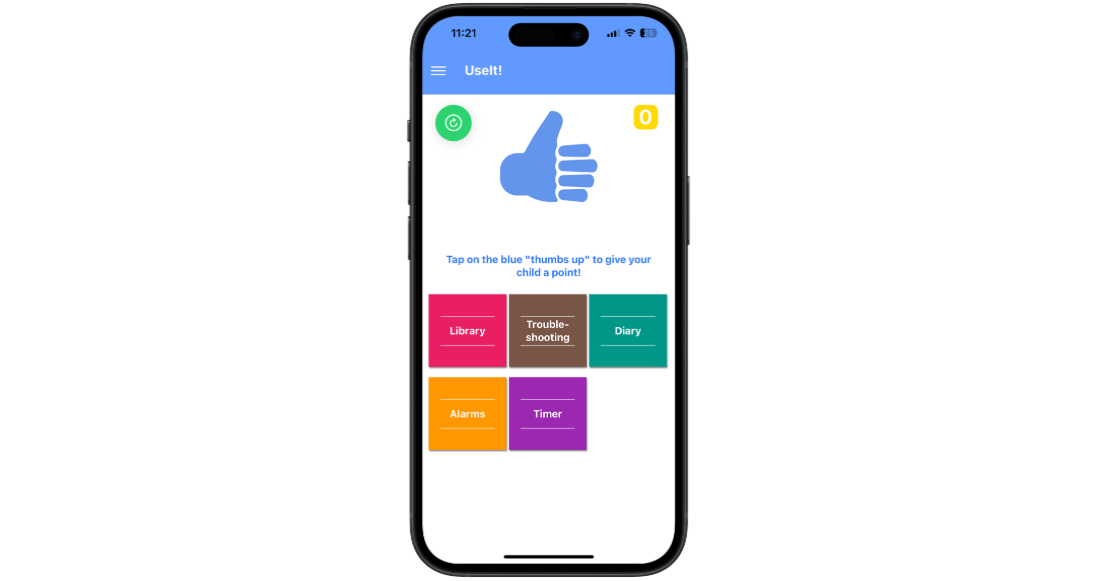
Home page of the UseIt! smartphone app.

The UseIt! system includes a free cross-platform mHealth app that runs on both iOS and Android devices. The app is securely connected to a portal where app feature usage is stored. The portal was designed for the research team to track and monitor the usage of the app by parents. The app contains six features: (1) a troubleshooting guide that provides detailed skill recommendations for problem situations, (2) a behavior diary for tracking behaviors and skills used each day, (3) a digital library that provides definitions and instructions for each skill, (4) a point counter for parents to award points to their children, (5) a skills-alarm for reminding parents to practice the various skills, and (6) a timer for use with parenting skills (eg, time-outs, managing screen time, routines). Users can examine diary entries, viewpoints awarded, and set the skills alarm through the app. See Figure 1 for a screenshot of the app’s home page.

#### Troubleshooting Guide

**Figure 2:**
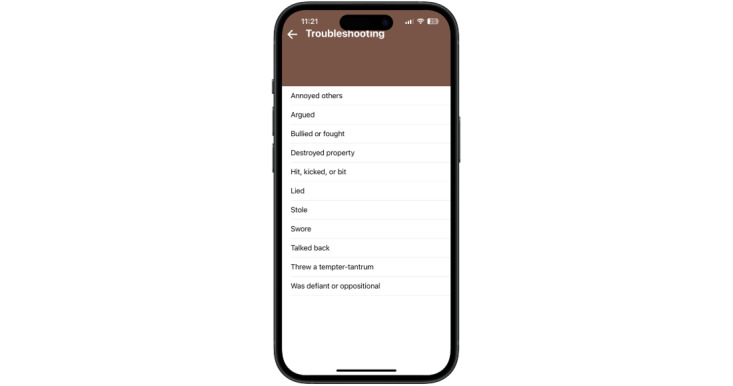
Troubleshooting guide.

The UseIt! troubleshooting guide contains information to help parents effectively respond to problem behaviors. Parents are presented with a list of potential negative behaviors (eg, bullied or fought). After selecting a behavior, appropriate skill options (eg, time-out) are displayed with tips to effectively apply each skill. Once a skill has been used and the behavior has stopped, parents are reminded to praise their child for positive behaviors (Figure 2).

#### Behavior Diary

**Figure 3:**
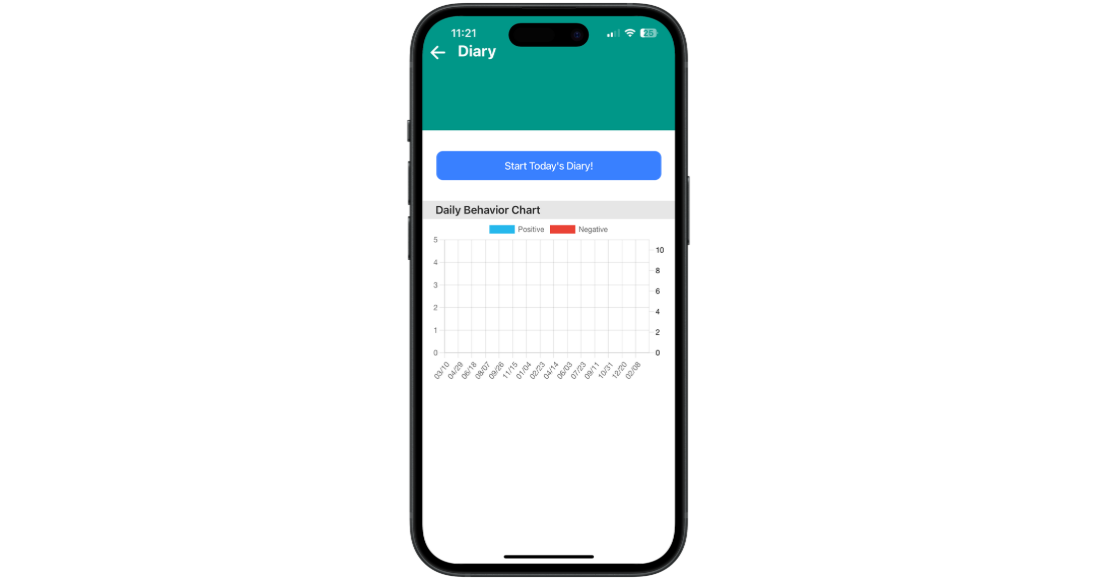
Daily behavior diary.

The behavior diary cues participants (via a notification) to complete a series of questions about behaviors and PMT and CBT skills used each day. The results are displayed graphically and can be reviewed by the user to track progress over time. This allows the user to keep track of what skills the family has tried for different behaviors (both positive and negative child behaviors) and which ones have been helpful in various contexts (Figure 3).

#### Digital Library

**Figure 4:**
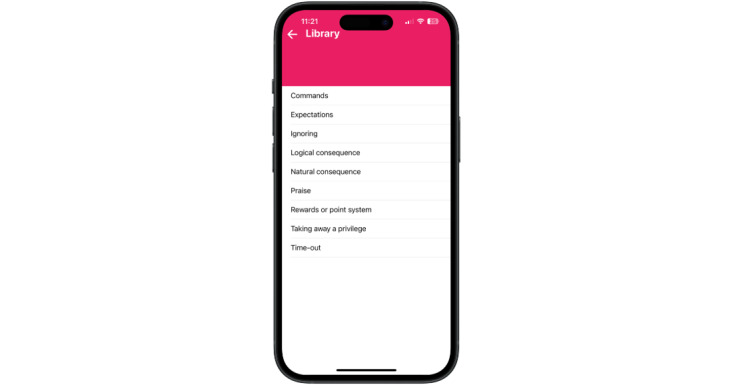
Digital library.

The UseIt! digital library provides detailed information about using strategies for positive and negative child behavior. Each skill is defined and presented with tips for how and when to effectively use each skill. The digital library contains more information than the troubleshooting guide and is designed as an information source for reviewing PMT and CBT topics (Figure 4).

#### Point Counter

Treatment for disruptive behavior disorders typically includes prizes for treatment adherence, positive behaviors, and skill use. Parents can award points to children for target behaviors (eg, cleaning dishes) and skill usage. The UseIt! point counter features an on-screen button (“Give your child a point”) which parents press to reward their child with a point. The feature functions as a digital rewards program that parents can use to keep track of points and reward their children. See Figure 1 for a view of the point counter window.

#### Skills Alarm

**Figure 5:**
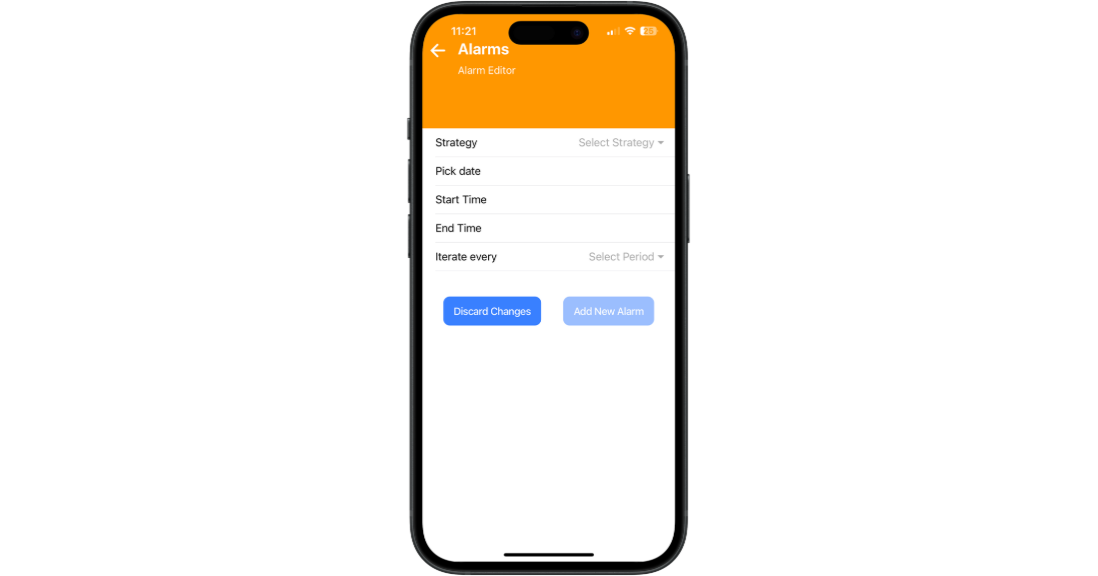
Skills alarm.

Skills alarms can be set at any time via the app. Users can set dates and times for notifications to activate. These notifications remind parents and children to practice specific skills throughout the week (eg, “remember to praise your child”). Parents are able to view a list of active and inactive alarms (Figure 5).

#### Timer

Timers can be set for use with a variety of skills (eg, time-outs, screen time management, and routines) to promote use of the skills.

### Recruitment

We use a 2-pronged recruitment strategy to maximize enrollment. The two recruitment avenues are (1) the Clinical and Translational Science Institute patient registry (Pitt+Me) at the University of Pittsburgh and (2) BuildClinical. The Clinical and Translational Science Institute patient registry (Pitt+Me) is an institutional research participant registry that uses enhanced study descriptions and social media to engage the community in research. BuildClinical is a clinical trial recruiting system that helps investigators recruit participants for clinical trials more efficiently. Using study-specific digital advertisements displayed on search engines, health websites, and social media platforms, BuildClinical generates participant referrals. BuildClinical also provides tools to streamline the recruitment and prescreening process. The platform stores information in a HIPAA (Health Insurance Portability and Accountability Act)-compliant manner and allows for remote enrollment.

### Participant Eligibility

Inclusion criteria for this study are that (1) parents or guardians must have a child between the ages of 5 and 8 years, (2) the child must be above the 90th percentile for either or both ODD and CD on the Vanderbilt Assessment Scale, (3) the child must be in residence with the parent or guardian for at least 80% of the time, (4) the parent or guardian must consent to study participation, and (5) the parent or guardian must have a smartphone device with daily internet access. Exclusion criteria are the child (1) having a known preexisting behavioral or mental health diagnosis requiring alternative treatment (eg, bipolar disorder, major depression, and pervasive developmental disorder) or (2) currently receiving treatment for childhood disruptive behavior.

### Sample Size Determination

Power analyses were conducted in PASS (version 13.0.8; NCSS) to ensure that the sample size is adequate to test the primary hypotheses with adequate statistical power. All analyses assumed 0.80 power and 2-sided tests. Estimated sample sizes are determined based on an assumed 5% attrition and missing data post treatment (102 per group) and 10% total attrition at 6-months (97 per group). Using an analysis of covariance approach to test for differences across 3 groups post treatment (n=108 each) and assuming *R*^2^=0.20 from 5 covariates, we expect 0.80 power (α=.05) to detect an effect size difference of Cohen *f*=0.16 among the 3 groups, *f*=0.14 (*d*=0.28) between both UseIt! treatment groups versus the control group, and *f*=0.18 (*d*=0.36) between any 2 groups.

### Design

The study is a RCT with 3 conditions. Parents of children aged 5 to 8 years (n=324) are randomly assigned to one of 3 conditions: a stand-alone UseIt! app condition, a UseIt! app and Coach condition, and a control app condition (Smiling Mind app). Data are collected via web-based surveys at baseline, post treatment (4 months after baseline), and at 6-month follow-up. Randomization takes place after the baseline is completed.

### Study Conditions

#### Stand-Alone UseIt! App Condition

Participants are assigned to the UseIt! App as a stand-alone intervention for 4 months.

#### UseIt! App + Coaching Condition

Participants are assigned to the UseIt! App and are provided with an mHealth “Coach” for 4 months. The primary objective of the coaching condition is to promote engagement with the UseIt! mHealth platform. The mHealth coach is a bachelor’s-level paraprofessional with a degree in psychology or an allied discipline (eg, social work) who provides support to parents in the coach-assisted condition. We selected a bachelor’s-level coach over a master’s-level coach to enhance the scalability of this intervention condition. The coach uses the SA coaching model using the training guidelines outlined by Dopke and colleagues [26]. During the intervention phase of the study, parents are contacted by the coach once per week by phone and also allowed to contact the coach during regular business hours. The coach provides motivation and accountability but does not provide therapeutic or clinical support. The primary goal of the coach is to increase participant engagement with the UseIt! mHealth system. Specific coaching content and tasks include (1) social support, (2) promoting engagement with the app, (3) goal setting (4) monitoring progress, and (5) encouragement and motivation. Parents are provided with appropriate referrals for any crises. The coach is instructed to respond to questions that lie outside the domains of motivation and accountability (ie, content-specific therapeutic support) by redirecting the parent to the content-specific app features (ie, Troubleshooting and Library). Only the parents interact with the coach. To maintain the scalability of the condition, the target time spent with each family is 15-30 minutes per week. The coach maintains a “coach-log” to track the frequency, duration, and content of contact with each participant.

#### Control Condition (Smiling Mind App)

Parents in the control app condition are assigned to use a mindfulness app called Smiling Mind [27] for 4 months. We selected a mindfulness app because it is an active control condition, but one that we do not expect to engage the same treatment targets as the UseIt! mHealth system. Meta-analytic findings indicate that mindfulness-based interventions for school-age children are associated with medium to large effect sizes for disruptive behaviors [28]. For parents, dispositional mindfulness has been found to be associated with lower rates of children’s externalizing and internalizing problems [29], and a mindfulness-based program was associated with decreased parent reports of child attention-deficit/hyperactivity disorder symptoms and decreased parental stress [30]. Other studies with youth found that a combined approach (parent and youth mindfulness training) improved externalizing problems and attention [31]. As an active control condition, we expect that a mindfulness app will likely have some influence on parenting and child behavior. This provides us with a rigorous control condition while also allowing us to test the specificity of target engagement. We expect the Smiling Mind app will only enhance mindfulness, but not PMT and CBT skills (other than mindfulness). We selected the Smiling Mind app [27] in particular because (1) it can be downloaded at no cost, (2) it is available for both Android and Apple (iPhone) devices, and (3) app-use can be tracked.

### Study Procedures and Randomization

Participants provide their contact information to the Pitt + Me or BuildClinical systems after accessing the study advertisement. Trained research assistants then contact families and conduct the initial screening to determine study eligibility. If determined eligible, a future call is scheduled to obtain consent from the parent, and assent from the child to participate. After consent, parents are provided a Qualtrics link to complete the initial baseline assessment. After completion of the assessment, families are randomly assigned to group 1 (standalone UseIt! app; n=108), group 2 (UseIt! app + coach; n=108), or group 3 (control app condition; n=108). We use stratified randomization to ensure that the groups are equivalent on key clinical features (screening severity and referral source (ie, Pitt + Me or BuildClinical). Parents assigned to the Coach condition are walked through the initial setup and login process, along with a brief training on how to use the applications over the phone. Families can be set up and trained in approximately 30 minutes. If parents cannot be reached after 3 weeks, instructions are sent via email. Parents assigned to the stand-alone UseIt! app condition are sent a tutorial video with the same information. Parents use the app condition assigned for 4 months before the administration of the posttreatment assessment, via Qualtrics. Six months following the posttreatment assessment, parents again are prompted to complete the 6-month follow-up assessment via Qualtrics.

### Measures

Table 1 for a summary of study constructs, measures, and assessment time points.

**Table 1:** Measures.

Construct	Measure or instrument	Time point
PMT^a^ and CBT^b^ skill knowledge	Knowledge of Effective Parenting Test	Baseline, post treatment, 6-month follow-up
Symptom severity	Vanderbilt Assessment Scale	Baseline, post treatment, 6-month follow-up
Parenting practices	Alabama Parenting Questionnaire	Baseline, post treatment, 6-month follow-up
Parent depression	7-item Generalized Anxiety Disorder Scale	Baseline, post treatment, 6-month follow-up
Parent anxiety	9-item Patient Health Questionnaire	Baseline, post treatment, 6-month follow-up
Parenting stress	Parental Stress Scale	Baseline, post treatment, 6-month follow-up
Social support	Social Provisions Scale	Baseline, post treatment, 6-month follow-up
PMT and CBT skill use	Parenting Skill Use Diary	Baseline, post treatment, 6-month follow-up
App use	Automatically recorded	Active app phase
Mindfulness	Mindful Attention Awareness Scale	Baseline, post treatment, 6-month follow-up
Service use	Service Assessment for Children and Adolescents	6-month follow-up
Supportive accountability	Supportive Accountability Inventory	Post treatment (coach condition only)
Usability	Post-Study Usability Questionnaire	Post treatment
Technological literacy	Technology Self-Assessment Tool	Baseline

^a^PMT: parent management training.

^b^CBT: cognitive behavioral therapy.

### Primary Outcome: PMT and CBT Skill Knowledge

The knowledge of effective parenting test [32] is a 21-item measure of parental knowledge of effective parenting skills. The measure was developed as a potential treatment target for evidence-based psychosocial treatments of disruptive behaviors in children. The knowledge of effective parenting test assesses parental knowledge of domains including praise, rewards and point systems, attending and ignoring, commands and expectations, consequences, and time-outs. Parents are presented with a series of video and text-based parenting scenarios and questions with 4 multiple-choice response options. Scores range from 0 to 21. The measure has good reliability (Cronbach α=0.84). The measure has also demonstrated convergent validity with other measures of parenting knowledge and parenting-related constructs (eg, child behavior and parental psychopathology).

### Secondary Outcomes

#### Symptom Severity

The Vanderbilt Assessment Scale-Parent Report (VASPR [33]) is a 55-item parent-report screen for attention-deficit hyperactivity disorder (ADHD), ODD, and CD. It also includes 7 items on internalizing symptoms and 8 items on school performance and social functioning. Symptom items are rated using a 4-point scale and the performance items are rated on a 5-point scale. The measure has Cronbach alphas ranging from 0.79 to 0.95 and strong evidence of construct validity.

#### Parenting Practices

The Alabama Parenting Questionnaire (APQ [34]) is a 42-item measure that assesses five dimensions of parenting: (1) positive involvement, (2) monitoring, (3) positive discipline, (4) consistency, and (5) corporal punishment, using a 5-point scale ranging from 1 to 5. The internal consistency of the scale is acceptable with α values for the 5 domains ranging up to .80. The measure has well-established construct validity.

#### Parent Depression

The Patient Health Questionnaire-8 (PHQ-8 [35]) measures symptoms of depression using a 4-point scale from “not at all” to “nearly every day.” Total scores range from 0 to 24. The measure has a reported Cronbach α of .82 and strong construct validity.

#### Parent Anxiety

The General Anxiety Disorder-7 Scale (GAD-7 [36]) is a 7-item measure of anxiety. Items are rated on a 4-point scale from “not at all” to “nearly every day.” The measure includes an item to assess the duration of anxiety symptoms. The measure has excellent internal consistency (Cronbach α=0.92), good test-retest reliability (intraclass correlation [ICC]=0.83), and strong convergent validity with other measures of anxiety.

#### Parenting Stress

The Parental Stress Scale (PSS [37]) is an 18-item measure of stress related to parental experiences. Items are rated on a 5-point scale from “strongly disagree” to “strongly agree.” Scores range from 18 to 90. The internal consistency of the scale is acceptable with a Cronbach α of 0.83, a test-retest reliability of 0.81 (ICC), and strong convergent validity of both other parental stress measures (ie, Parental Stress Index), and other measures related to parenting stress (eg, loneliness, marital satisfaction, and social support).

#### Social Support

The Social Provisions Scale [38] is a 24-item measure that assesses 6 dimensions of support, including attachment, social integration, opportunity for nurturance, reassurance of worth, reliable alliance, and guidance. The measure uses a 5-point scale ranging from 1 to 4, strongly disagree to strongly agree. The measure has been validated across samples, with Cronbach α ranging from 0.65 to 0.76 for the 4 subscales, and a total reliability estimate of 0.91. The measure also demonstrated convergent validity with related measures of social support.

#### PMT and CBT Skill Use

The Parenting Skill Use Diary [39] assesses daily use of parenting skills in everyday parenting contexts (eg, child sharing and helping, hitting, and fighting). Respondents are presented with a checklist of behaviors to report on for the past week. For each behavior they select, they are next asked to identify which skills (eg, praise, reward, time-out, and loss-of-privilege) they used in responding to the behaviors. The instrument has demonstrated the ability to capture significant between-person variability in appropriate PMT skills. A weekly summary score discriminated between parents or guardians whose children screened positive versus negative for CD (area under the receiver operating characteristic curve [AUC]=0.72) and ODD (AUC=0.70).

#### App Use

Parents assigned to both UseIt! conditions (ie, stand-alone app and coach conditions) have their data stored on the secure portal, accessible to the research team. We collect the behavior diary tracked by parents, which displays data on child behavior (both positive and negative) along with CBT and PMT skills. We are also collecting data on time spent on the app along with modules accessed. App usage (eg, modules used and time spent) is also tracked for the Smiling Mind app (control condition).

#### Mindfulness

The Mindful Attention Awareness Scale [40] is a 15-item scale designed to assess core characteristics of mindfulness. Parents are asked how often they are engaging in a variety of mindfulness-related behaviors, such as finding it difficult to stay focused on what is happening in the present moment, finding themselves preoccupied with the future or past, and not noticing feelings of physical tension or discomfort until the really grab their attention. The measure’s response scale ranges from 1 (almost always) to 6 (almost never). The measure is scored as an average of all 15 items. The measure has adequate validity (Cronbach α=0.87 and convergent validity with other measures of mindfulness (eg, mood disturbances and stress).

#### Service Use

An abbreviated version of the Service Assessment for Children and Adolescents (SACA [41]; 25 items) was used to measure mental health service usage. The SACA asks about various inpatient and outpatient treatment services for mental or behavioral health problems that have been used by the child in the past 6 months [41]. Most items are yes or no questions. The measure is a widely used research tool with strong evidence of reliability and validity.

#### Supportive Accountability

The Supportive Accountability Index (SAI [42]) is an 8-item measure of how well a given platform functioned to help keep parents accountable to accomplish a given goal. The measure was included to assess the effectiveness of the Coach in the coach condition of the app in helping to keep parents accountable with skill learning and use. Items are rated on a 1-7 scale, from 1=strongly disagree to 7=strongly agree. Total scores are summed, ranging from 8-56. The measure has acceptable validity (Cronbach α=0.68) and good convergent and divergent validity.

#### Usability

The 19-item Post-Study System Usability Questionnaire (PSSUQ [43]) is used to assess overall user satisfaction with the UseIt! apps. Internal consistency of the PSSUQ is excellent (α=.91 to .96).

#### Technological Literacy

The Technological Self-Assessment Scale (TSAT [44]) is a 13-item parent report screen for technological ability. The measure was created for this study by the research team to provide an indicator of parent knowledge and experience with their computers and phones. For example, items ask parents if they know how to search for information on the internet if they have ever downloaded an app, and if they have social media accounts. Items are scored Yes or No, and scores range from 0 to 13.

### Data Analyses

#### Primary Analyses

Our primary analytic strategy will use intent-to-treat analyses. We will examine reasons for any missing data and perform multiple imputation (eg, Multiple Imputation for Chained Equations [45]) for data missing at random. To evaluate the effectiveness of the UseIt! mHealth system as both a stand-alone (n=108) and coach-assisted (n=108) intervention compared to a control app condition (n=108), we will regress the primary and secondary outcomes on the study condition. We will also perform a priori tests to compare both UseIt! groups to the control and to compare the coach-assisted and stand-alone UseIt! groups. Cohen *d* effect sizes will be estimated for between-group differences as well as pre-post changes within each group.

#### Secondary Analyses

We will also test whether gains in knowledge of parenting skills are associated with symptom reduction. We will regress the posttreatment knowledge of parenting skills on study condition, pretreatment knowledge, and their interaction. We will also compare app usage across stand-alone and coach-assisted conditions and test whether the app usage indices are associated with target engagement and symptom reduction at post-treatment. App usage outcomes will include the number of each of the app features that are used, along with frequency and duration (in minutes). We will use generalized linear models with the appropriate link (eg, log link for count data and identity link for continuous outcomes) to regress each usage outcome on study condition (stand-alone versus coach).

### Ethical Considerations

Ethical approval has been obtained from the institutional review board at the University of Pittsburgh (protocol # STUDY22030138). Informed consent from parents is obtained by trained research staff. So as not to artificially inflate the rates of app usage, participants are not compensated for using the app but only for assessments completed. Participants in each condition are compensated US $20 for baseline assessment completion, US $40 for the postassessment, and US $60 for the 6-month follow-up assessment. All study data are deidentified and stored securely.

## Results

**Figure 6:**
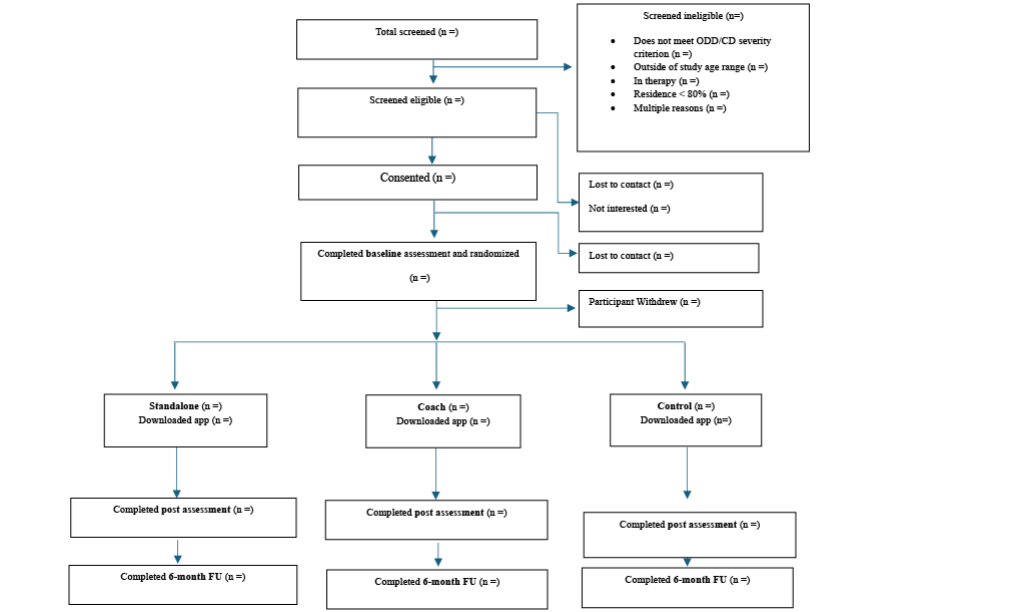
Trial flowchart. FU: follow-up.

The study was funded in September 2022. Study recruitment began in December 2022. As of December 2024, we have recruited over half (n=214) of our target sample of 324 parent-child dyads. Of these, 72 parents have been assigned to the stand-alone condition, 71 to the coach condition, and 71 to the control condition. Recruitment is ongoing and completion is expected in another 12 to 14 months. Follow-up data collection is expected to be completed by the end of 2026. Figure 6 displays the trial flowchart for this study.

## Discussion

mHealth parenting programs have the potential to improve outcomes for parents and children [14]. Further, supportive accountability, defined as a degree of human interaction throughout the program, has the potential to enhance outcomes for mHealth interventions by increasing motivation and engagement [23]. The current study aims to add to the literature base on effective mHealth interventions to treat disruptive behaviors in children. The study will allow us to test the degree to which the UseIt! app can modify parenting (ie, skill acquisition and usage) and whether such target engagement is associated with symptom reduction. The UseIt! mHealth system also allows researchers unique access to data on child behaviors and parent skills tracked on a daily basis. This data will not only allow parents to track behaviors and skills for themselves (allowing parents to visualize progress) but will also allow researchers a system to track change over time. The coaching model used in the study is highly scalable, providing a flexible model of coaching that can be tailored to a variety of clinical conditions and service settings. We expect that those assigned to the coach condition will have the largest increases in parent knowledge and skill use, along with the largest decreases in child behavior problems. We also expect that those assigned to the UseIt! stand-alone app condition will report greater improvements in parent knowledge, skill use, and child behavioral concerns, compared with the control app condition.

### Strengths and Limitations

This study has several strengths and adds to the existing literature. To our knowledge, it is the largest RCT of an mHealth smartphone-based app targeting child behavioral concerns to date. The trial adds to the literature base of the SA model [23] and aims to provide preliminary evidence to the effectiveness of the model in the context of a smartphone-based app targeting parents. We selected clinically relevant outcome measures for the trial, including validated measures of parenting knowledge, parent skill use, parent mental health, and child behavior problems. We also collect app usage data, both for use as a moderator of intervention effects, and to inform future work to promote increased usage in other studies. A few limitations are also worth noting. First, we expect up to 10% attrition over the course of the study. We have taken several steps to minimize attrition, including increasing participant compensation at each time point. We also factored 10% attrition into our power analysis to determine sample size, ensuring that the trial will still be fully powered. Another limitation is that outcome measures are based primarily on parent report. This limitation is mitigated by substantial evidence that parents are accurate reporters of child behavior problems.

### Conclusions and Future Directions

In summary, the current study aims to address a gap in the literature regarding the feasibility, effectiveness, and utility of a smartphone app that includes a coach-assisted arm for treating disruptive behaviors in young children. Digital therapeutics have the potential to enhance the reach and scalability of skills-based psychosocial interventions, as even small effects can be meaningful on a population level if the intervention can be delivered efficiently on a large scale at a low cost. The UseIt! mHealth system is able to deliver therapeutic content to parents across a variety of settings and has the potential for meaningful impact. Findings from the current trial will advance scientific knowledge and have the potential to enhance clinical practice.

## Appendix

Multimedia Appendix 1 Peer-review report from CPDD - Child Psychopathology and Developmental Disabilities Study Section (National Institutes of Health, USA).

Multimedia Appendix 2 CONSORT-eHEALTH checklist (V 1.6.1).

Multimedia Appendix 3 Spirit Checklist.

## Abbreviations

ADHD: attention-deficit/hyperactivity disorder
APQ: Alabama Parenting Questionnaire
AUC: area under the receiver operating characteristic curve
CBT: cognitive behavioral therapy
CD: conduct disorder
DBD: disruptive behavior disorder
DSM-5: Diagnostic and Statistical Manual of Mental Disorders (Fifth Edition)
GAD-7: General Anxiety Disorder-7 Scale
HIPAA: Health Insurance Portability and Accountability Act
mHealth: mobile health
ODD: oppositional defiant disorder
PHQ-8: Patient Health Questionnaire-8
PMT: parent management training
PSS: Parental Stress Scale
PSSUQ: Post-Study System Usability Questionnaire
RCT: randomized controlled trial
SA: Supportive Accountability
SACA: Service Assessment for Children and Adolescents
SAI: Supportive Accountability Index
TSAT: Technological Self-Assessment Scale
VASPR: Vanderbilt Assessment Scale-Parent Report
