# Supplementary material for: Novel Smartphone App and Supportive Accountability for the Treatment of Childhood Disruptive Behavior Problems: Protocol for a Randomized Controlled Trial
Source: JMIR Res Protoc. 2025 Mar 11;14:e67051. doi: 10.2196/67051 (PMC11937717; doi:10.2196/67051)
Supplement: Multimedia Appendix 1 [file resprot_v14i1e67051_app1.pdf]

1R01HD106930-01A1 LINDHIEM, OLIVER

NEW INVESTIGATOR

RESUME AND SUMMARY OF DISCUSSION: The objective of this this revised application is to test the effectiveness of a mobile heath application, “UseIt!”, an intervention designed for parents of children ages 5-8 years old with disruptive behavior disorders (DBD). The project will compare use of the UseIt! app alone compared to UseIt! in combination with a “coach” and both compared to a control condition (mindfulness app). In addition, there will be examination of the effectiveness of individual components of UseIt!. There was universal enthusiasm among the panel for the significance of proposed work in that, if successful, the project will improve the scalability and reach of administering evidence-based treatment for DBDs by reducing critical barriers. Further, the work has a strong foundation and builds logically from the team’s prior work. The application was highly responsive to prior review which strengthened the project. The investigative team was considered very strong with expertise in the treatment of behavioral disorders, CBT and digital therapeutics and the appropriate statistical knowledge to execute the data analytic plan. Further, the history of prior successful collaborations was a strength. Using coaching and mobile technology to teach parents intervention strategies for DBDs and comparing the two active conditions was considered innovative. Numerous methodological strengths were noted including use of a large national sample, longitudinal design, strong recruitment plan and focused recruitment of under-represented minorities and females with DBDs. Additionally, use of a control condition and examination of the individualized components of the application will allow for a “personalized medicine” approach to tailoring individualized treatment. A relatively minor weakness in the approach included limited training for the use of CBT and parent management skills outside the application which can be difficult for some parents; however, given strong preliminary data on this issue and the ability to examine this issue in the current design, this was considered a minor weakness. Further, the training for the app occurs during a single 30-minute session which may not be enough to assess parental comprehension of the app and skills. Following the discussion, the concerns only slightly diminished the overall high potential impact of the proposed work on the field of DBD treatment.

DESCRIPTION (provided by applicant): Although evidence-based treatments (EBTs) have been developed for childhood behavior problems, many families do not have access to these services. Barriers to access include local availability of services, transportation, cost, and perceived stigma. In this project, we aim to test the effectiveness of a mobile health (mHealth) system as a standalone versus coach-assisted intervention with the goal of achieving reach and scalability. The project will consist of an RCT to study the effectiveness of an mHealth system called UseIt! (Utilizing Skills to Enhance the Impact of Treatment) to deliver treatment remotely and promote the use of parent management training (PMT) skills and cognitive-behavioral therapy (CBT) skills. UseIt! is part of the growing field of digital therapeutics, based on the concept that electronic devices can enhance health outcomes and reduce costs. Parents of children (ages 5-8) with disruptive behaviors (N = 324 dyads) will be randomly assigned to Group 1 (standalone app; n = 108), Group 2 (coach-assisted app; n = 108), or Group 3 (control app; n = 108). The outcome assessments (post-treatment and 6-month follow-up) will include measures of target engagement (PMT/CBT skill acquisition and utilization) and symptom reduction. This RCT takes an experimental therapeutics approach and is designed to measure target engagement to determine whether target engagement is associated with symptom reduction. The targets are PMT/CBT skill acquisition and utilization. Skill deficits in these domains are understood as key maintaining factors for serious disruptive behavior problems. Aim 1 is to evaluate the effectiveness of the UseIt! mHealth system as both a standalone and coach-assisted intervention compared to a control app condition. Aim 2 is to assess target (PMT/CBT skill acquisition and utilization) engagement and validation. Aim 3 is to evaluate the effectiveness of the components of the UseIt! mHealth system. The project has the potential to make an impact on both scientific knowledge and clinical practice. In terms of scientific knowledge, the project has the potential to expand our understanding of target mechanisms. By incorporating reliable and valid measures of the treatment

targets, the project will be informative regardless of the clinical outcome. In terms of clinical practice, digital therapeutics has the potential to enhance the reach and scalability of skills-based psychosocial interventions. Even small effects can be meaningful on a population level if the intervention can be delivered easily on a large scale at low cost. The project is consistent with NICHD's mission to ensure "that all children have the chance to achieve their full potential for healthy and productive lives."

PUBLIC HEALTH RELEVANCE: This clinical trial will test the effectiveness of a smartphone-based mobile health (mHealth) system called UseIt! as both a standalone and coach-assisted intervention. The UseIt! mHealth system includes both an app and online portal and is designed to work by promoting skills-practice. The project has the potential to increase access to care by boosting the reach and scalability of skills-based psychosocial interventions for childhood behavior problems.

CRITIQUE 1

Significance: 2
Investigator(s): 1
Innovation: 3
Approach: 3
Environment: 1

Overall Impact: This randomized control trial builds upon prior work by the PI and study team by evaluating the effectiveness of an mHealth app called, "UseIt!". UseIt! is designed to train parents of youth ages 5-8 years with DBDS in the use and application of CBT and PMT skills. The novel element of the present application is a comparison of the UseIt! app alone to the UseIt! app in combination with a "coach" to a mindfulness app which serves as a control condition, as well as the examination of the effectiveness of the individual components of the UseIt! app in a large, nationwide sample and via a longitudinal design. The project's significance is that it has the potential to provide increased scalability and a wider reach of empirically supported interventions for youth with DBD, of which there are over 1.5 million youth in the US. The team of researchers have a history of collaboration spanning over a decade on projects spanning treatment delivery for youth with a variety of behavioral health concerns, treatment of DBD specifically, CBT, mindfulness, and mHealth interventions. Further, the environment has adequate resources to support both recruitment of participants and to support the work of the team. With respect to weaknesses, relatively minor weaknesses were observed with respect to approach and innovation. Specifically, with respect to innovation, much prior work by the team has examined the effectiveness of the app, and the addition of the "coach" condition is an appropriately incremental and logical step. With respect to approach, training appears to be limited to 30 minutes and limited to instruction in how to use the app. There appears to be no instruction (outside of the app) with respect to CBT and PMT skills, which can be challenging for some parents to master. Finally, while the effort to recruit 30% underrepresented minorities is commendable, as of 2019, roughly 40-45% of individuals in the USA identify as such. Thus, recruitment of underrepresented minority youth at 30% of the sample should represent a minimum. In sum, the project, if successful, has the potential for a significant and relatively high impact to improve the accessibility of interventions for youth with DBDs.

1. Significance:

Strengths

- The project addresses a significant public health concern, as over 1.5 million youth meet diagnostic criteria for DBDs. Without effective intervention, youth with DBDs may develop more severe externalizing concerns and/or internalizing concerns.
- The project utilizes mHealth to address and reduce several barriers that reduce access to effective treatments, particularly with respect to proximity to qualified providers of effective

interventions. The design allows for an examination of whether brief supplemental coaching improves outcomes over and above the mHealth approach.

- The project utilizes existing evidence based techniques via the incorporation of PMT and CBT skills, which have been shown to reduce child behavior problems.

Weaknesses

- No weaknesses noted.

2. Investigator(s):

Strengths

- The proposed team has a history of successful collaboration in projects that served as the basis for the present proposal spanning over a decade, resulting in numerous grants, conference presentation, and published manuscripts.
- PI Dr. Lindheim has experience via both prior funding and published manuscripts in the areas of assessment of CBT and PMT skill acquisition and utilization among parents. Dr. Lindheim has also worked via prior pilot funds to develop and examine the effectiveness of the mHealth technology being used. Dr. Lindheim also has extensive history in the treatment of DBDs in youth.
- Co-I Dr. Hafeman has experience in the area of the utilization of apps to provide mindfulness interventions (i.e., the control condition) for individuals with mood disorders, and has prior experience in the domain of examining predictors of treatment effectiveness.
- Co-I Dr. Parmanto has experience in the domain of developing tele/mobile interventions and has been integral in the development of the UseIt! system.
- Co-I Kolko has extensive experience in the assessment and treatment of child disruptive behavior problems, and has worked closely with the team in the development and evaluation of the UseIt! app.
- Co-I Silk developed an app to improve CBT treatment for youth.
- Co-I Dr. Wallace is a statistician who is well-versed in analytic methods to examine clinical outcomes and change.

Weaknesses

- No weaknesses noted.

3. Innovation:

Strengths

- The development, refinement, and subsequent dissemination of a mobile app to provide parents with the ability to acquire and practice PMT and CBT skills is innovative, while addressing the need for scalability and wide reach of services for youth with DBDs.
- An examination of which components of the UseIt! app provide the greatest benefit will allow for further refinement.
- The clinical trial aspect pitting the UseIt! app against the UseIt! app plus coaching against a control app will provide insight into the necessary and sufficient elements required for such mHealth interventions.

Weaknesses

- Prior work of the PI and co-I team has served to validate the UseIt! app across several studies; however, these prior studies utilized relatively small samples. The addition of the add-on coach for 15-30 minutes per week seems like a relatively small step. Particularly, given that prior work described in the Background and Significance sections suggest “some degree of human interaction is important to achieve and sustain meaningful outcomes in mHealth interventions.” It is unclear why or how UseIt! would or would not be expected to differ in this regard.

4. Approach:

Strengths

- The target sample of youth between ages 5-8 is appropriate and represents an important age for intervention in the context of DBDs, as prior work shows they may reduce the risk of escalation of future affective and behavioral concerns across development.
- The control group in addition to the two targeted intervention groups (i.e., UseIt! and UseIt! plus coach) represents an appropriate design to examine the effectiveness, as well as the necessary and sufficient components of a mHealth intervention for youth with DBD.
- The study builds on prior work that has served to validate the UseIt! app.
- The use of three separate recruitment pipelines, particularly Trialspark, which has the ability to reach the target population of parents of youth ages 5-8, as well as parents of youth of both genders and parents of minority youth is a strength, as it will allow for the recruitment of a large and generalizable sample.
- The follow-up/”exploratory” analyses targeting the effects of individual app components, child behaviors, and parental engagement will allow for further refinement of the app in future endeavors.
- The study provides participants with data plans when needed allowing for greater inclusion.

Weaknesses

- It appears that training occurs over the course of a single 30-minute phone session. While the app itself may be intuitive and easy to learn in that period. Clinically, many parents struggle with understanding and/or applying the PMT and CBT skills in the hands of a skilled clinician. This is also true of mindfulness-based approaches and skills. It is unclear whether there are checks for comprehension. It appears that addressing questions related to understanding or applying skills is outside of the scope of the “coach” role.

5. Environment:

Strengths

- The environment (i.e., University of Pittsburgh, and associated School of Medicine and Department of Psychiatry, as well as UPMC Western Psych) is well-suited to support the research, providing all necessary resources, including all office and clinic space, local recruitment pipelines, office of grants and contracts to assist with administration, computing facilities, as well as access to the larger recruitment system (i.e, Trialspark).

Weaknesses

- No weaknesses noted.

Study Timeline:

Strengths

- Timeline is appropriate to complete the scope of work.

Weaknesses

- No weaknesses noted.

Protections for Human Subjects:

Acceptable Risks and/or Adequate Protections

- Appropriate protections for human subjects are in place.

Data and Safety Monitoring Plan (Applicable for Clinical Trials Only):

Acceptable

- An appropriate data and safety monitoring plan has been proposed for the level of risk associated with this project.

Inclusion Plans:

- Sex/Gender: Distribution justified scientifically
- Race/Ethnicity: Distribution justified scientifically
- For NIH-Defined Phase III trials, Plans for valid design and analysis: Not applicable
- Inclusion/Exclusion Based on Age: Distribution justified scientifically
- Sex distribution is appropriate with a slight oversampling of male youth, given the prevalence of DBDs. Ethnic and racial minority youth are anticipated to make up roughly 30% of the sample--as of 2019 roughly 60% of the US population identified as white and non-Hispanic/Latinx, as such an effort to include at least 30% ethic/racial minority participants is justified, but could be expanded. Including youth between ages 5 to 8 years is scientifically justified and a typical age of onset for DBD concerns.

Resubmission:

- The primary issues raised in prior reviews (including: providing clarity related to the "coach" role/qualifications/training, ability to recruit, retain, and work with a diverse sample, recruitment of youth with DBD concerns, analytic plans, data plans, etc.) have largely been addressed in the Response to Reviewers and associated sections. However, the issue related to app training remains, as an explanation related to the use of the app was provided, but little was stated regarding training in the content of the app with respect to understanding of CBT and PMT concepts and the application of those concepts.

Resource Sharing Plans:

Acceptable

Budget and Period of Support:

Recommend as Requested

CRITIQUE 2

Significance: 1
Investigator(s): 1
Innovation: 1
Approach: 2
Environment: 1

Overall Impact: This is an outstanding resubmission of an R01 application which proposes to test the effectiveness of a mobile health (mHealth) system as a standalone versus coach-assisted intervention with the goal of achieving reach and scalability. The project will consist of an RCT to study the effectiveness of an mHealth system called UseIt! (Utilizing Skills to Enhance the Impact of Treatment) to deliver treatment remotely and promote the use of parent management training (PMT) skills and cognitive-behavioral therapy (CBT) skills. Parents of children (ages 5-8) with disruptive behaviors (N = 324 dyads) will be randomly assigned to Group 1 (standalone app; n = 108), Group 2 (coach-assisted app; n = 108), or Group 3 (control app; n = 108). The outcome assessments (post-treatment and 6-month follow-up) will include measures of target engagement (PMT/CBT skill acquisition and utilization) and symptom reduction. This RCT takes an experimental therapeutics approach and is designed to measure target engagement to determine whether target engagement is associated with symptom reduction.

The team has been very responsive to prior reviews in this resubmission. Strengths from the original application remain: An outstanding investigative team with a history of working together, capitalizing on mobile technology to increase accessibility of services, preliminary research supports efficacy of the mobile app, the environment is outstanding, the recruitment strategy ensures minority representation. In this resubmission, the investigators address prior concerns around the coaches, training of the coaches, whether parents need to be trained in using the app, and attrition. Investigators also provide unlimited data plans to families who have limited plans. Remaining weakness is minor and surrounds whether there are checks on appropriate use of the app. Overall, strengths clearly outweigh weaknesses and impact is thought to be high.

1. Significance:

Strengths

- Disruptive Behavior Disorders (DBD) affect 1.5 million school-aged children. If left untreated, DBDs are associated with poorer outcomes (behavior, psychological, academic, and professional) later in life.
- Effective treatments for DBD exist; however, there are disparities in who has access.
- Capitalizing on parent management training and CBT skills via widespread mobile technology might decrease disparities.
- The targets are PMT/CBT skill acquisition and utilization because skill deficits in these domains are recognized as key maintaining factors for serious disruptive behavior problems.

Weaknesses

- None noted.

2. Investigator(s):

Strengths

- Dr. Lindhiem is clearly suited to be the PI of this project and has assembled an outstanding team. He has a strong history of funding in this area (i.e., parent skill acquisition), developed the mHealth technology used in this proposal, and has tested the effectiveness of the UseIt! technology.
- Members of the investigative team have a history of collaboration and bring expertise in: treatment of behavior disorders, CBT and digital therapeutics, the mindfulness app that will be used for the control condition, statistical analysis. Some have also been involved in the implementation of UseIt!

Weaknesses

- None noted.

3. Innovation:

Strengths

- Using coaching and mobile technology to teach parents/caregivers management skills and CBT skills is novel.

Weaknesses

- None noted

4. Approach:

Strengths

- Preliminary studies support the conceptual model driving this project.
- Control condition – mindfulness app – provides an active control condition that will allow a test of the specificity of target engagement.
- Coaching affords benefits of human interaction at a level of service that remains highly scalable. Coaches are trained by the PI.
- Purposive sampling will ensure 40% female and 30% URM participants. Related - TrailSpark – one arm of the recruitment strategy - has experience and capacity recruiting for behavioral health disorders and URM participants.
- Evidence to suggest that parents are able to use the app without additional training – lending to success and scalability.
- Assessments a baseline, post-treatment, and 6-month follow-up are conducted electronically and assess symptom reduction as well as contextual factors.
- Unlimited data plans will be provided to those families who have limited data plans.

Weaknesses

- While the investigators state that pilot work supports the ease of use (i.e., no parent training), it is unclear whether there are checks in place to ensure appropriate usage.

5. Environment:

Strengths

- The environment at all sites appears excellent.

Weaknesses

- None noted.

Study Timeline:

Strengths

- Timeline is appropriate for proposed work.

Weaknesses

- None noted

Protections for Human Subjects:

Acceptable Risks and/or Adequate Protections

- Appropriate protections in place

Data and Safety Monitoring Plan (Applicable for Clinical Trials Only):

Acceptable

- The DSM plan in place is appropriate for the proposed project.

Inclusion Plans:

- Sex/Gender: Distribution justified scientifically
- Race/Ethnicity: Distribution justified scientifically
- For NIH-Defined Phase III trials, Plans for valid design and analysis:
- Inclusion/Exclusion Based on Age: Distribution justified scientifically
- The proposed research will involve parents of children ages 5-8. Intervening with this age group is particularly important because childhood-onset conduct problems are more likely to persist into adulthood than adolescent onset conduct problems. At least 40% will be female. At least 30% will be URM.

Resubmission:

- This resubmission has been very responsive to prior reviews.

Resource Sharing Plans:

Acceptable

Budget and Period of Support:

Recommend as Requested

CRITIQUE 3

Significance: 1
Investigator(s): 1
Innovation: 2

Approach: 2
Environment: 1

Overall Impact: This revised application is designed to conduct a nationwide randomized controlled trial (RCT) of the UseIt! mHealth system among parents of children ages 5-8 years old. Parents of youth with disruptive behaviors (*N* = 324 dyads) will be randomized to one of three groups n = 108/group: standalone app, coach-assisted app, or an active control app (Smiling Mind mindfulness training). The study will assess validated clinical outcomes specific to parenting-based interventions, user satisfaction, and engagement of treatment targets. The study builds upon three NIH-funded projects and an award from the Klingenstein Foundation. The project includes a stellar team of researchers with expertise in optimizing treatment delivery for child/adolescent behavioral health disorders, treatment of disruptive behavior disorders, cognitive behavioral therapy, app-based treatment development, mindfulness, information management, bioinformatics, data mining, and biostatistics for personalized medicine. The University of Pittsburg is a well-resourced environment that is well suited for the proposed research. The study is appropriately incremental, building off of prior research with novel additions that enhance the innovation of the project. The study is well designed and, if successful, could contribute significantly to the reduction of disruptive behavior problems on a broad scale. The app-based approach is scalable and will also enhance reach for families who would otherwise not have access to evidence-based care. The recruitment strategy also appropriately samples for girls and underrepresented minorities, which will increase impact and generalizability. The revised application has several improvements that strengthen the proposed research. Specifically, the team has clarified their experience working with diverse populations. They have also increased the description of TrialSpark, which will be used for nationwide recruitment. In spite of this description, there is not very much information on the nationwide aspect of the study. For example, coaches can be contacted during business hours, but it is unclear whether those hours differ across time zones. Given that TrialSpark is only one of three recruitment methods, recruitment still seems biased toward participants in the Pittsburgh area, and it is unclear how geographically diverse the final sample will be. Nonetheless, this is an important study with potential to have a high impact on the field.

Study Timeline:

Strengths

- The timeline is appropriate.

Weaknesses

- No concerns.

Protections for Human Subjects:

Acceptable Risks and/or Adequate Protections

- Acceptable

Data and Safety Monitoring Plan (Applicable for Clinical Trials Only):

Acceptable

- This is a low-risk trial with an appropriate plan for unanticipated risks.

Inclusion Plans:

- Sex/Gender: Distribution justified scientifically
- Race/Ethnicity: Distribution justified scientifically

- For NIH-Defined Phase III trials, Plans for valid design and analysis: Not applicable
- Inclusion/Exclusion Based on Age: Distribution justified scientifically
- Acceptable

Resubmission:

- Responsive to prior review.

Resource Sharing Plans:

Acceptable

Budget and Period of Support:

Recommend as Requested

THE FOLLOWING SECTIONS WERE PREPARED BY THE SCIENTIFIC REVIEW OFFICER TO SUMMARIZE THE OUTCOME OF DISCUSSIONS OF THE REVIEW COMMITTEE, OR REVIEWERS' WRITTEN CRITIQUES, ON THE FOLLOWING ISSUES:

PROTECTION OF HUMAN SUBJECTS: ACCEPTABLE

INCLUSION OF WOMEN PLAN: ACCEPTABLE

INCLUSION OF MINORITIES PLAN: ACCEPTABLE

INCLUSION ACROSS THE LIFESPAN: ACCEPTABLE

COMMITTEE BUDGET RECOMMENDATIONS: The budget was recommended as requested.

Footnotes for 1 R01 HD106930-01A1; PI Name: LINDHIEM, OLIVER JAMES

NIH has modified its policy regarding the receipt of resubmissions (amended applications).See Guide Notice NOT-OD-18-197 at https://grants.nih.gov/grants/guide/notice-files/NOT-OD-18-197.html. The impact/priority score is calculated after discussion of an application by averaging the overall scores (1-9) given by all voting reviewers on the committee and multiplying by 10. The criterion scores are submitted prior to the meeting by the individual reviewers assigned to an application, and are not discussed specifically at the review meeting or calculated into the overall impact score. Some applications also receive a percentile ranking. For details on the review process, see http://grants.nih.gov/grants/peer_review_process.htm#scoring.

MEETING ROSTER

Child Psychopathology and Developmental Disabilities Study Section
Biobehavioral and Behavioral Processes Integrated Review Group
CENTER FOR SCIENTIFIC REVIEW
CPDD
02/22/2022 - 02/23/2022

Notice of NIH Policy to All Applicants: Meeting rosters are provided for information purposes only. Applicant investigators and institutional officials must not communicate directly with study section members about an application before or after the review. Failure to observe this policy will create a serious breach of integrity in the peer review process, and may lead to actions outlined in NOT-OD-14-073 at https://grants.nih.gov/grants/guide/notice-files/NOT-OD-14-073.html, NOT-OD-15-106 at https://grants.nih.gov/grants/guide/notice-files/NOT-OD-15-106.html, and NOT-OD-18-115 at https://grants.nih.gov/grants/guide/notice-files/NOT-OD-18-115.html, including removal of the application from immediate review.

CHAIRPERSON(S)

CORBETT, BLYTHE ANNE, PHD
PROFESSOR
DEPARTMENT OF PSYCHIATRY AND
BEHAVIORAL SCIENCES
VANDERBILT UNIVERSITY SCHOOL OF MEDICINE
NASHVILLE, TN 37203

MEMBERS

BRESS, JENNIFER N, PHD *
ASSISTANT PROFESSOR
DEPARTMENT OF PSYCHIATRY
WEIL CORNELL MEDICINE
NEW YORK, NY 10605

CAI, WEIDONG, PHD *
CLINICAL ASSISTANT PROFESSOR
DEPARTMENT OF PSYCHIATRY
AND BEHAVIORAL SCIENCES
STANDFORD UNIVERSITY
STANFORD, CA 94305

COFFEY, BARBARA JANE, MD
PROFESSOR
DEPARTMENT OF PSYCHIATRY AND
BEHAVIORAL SCIENCES
UNIVERSITY OF MIAMI
MIAMI, FL 33136

CROWELL, SHEILA E, PHD
ASSOCIATE PROFESSOR
DEPARTMENT OF PSYCHOLOGY
UNIVERSITY OF UTAH
SALT LAKE CITY, UT 84112

CULLEN, KATHRYN REGAN, MD
ASSOCIATE PROFESSOR
DEPARTMENT OF PSYCHIATRY
AND BEHAVIORAL SCIENCES
UNIVERSITY OF MINNESOTA
MINNEAPOLIS, MN 55454

DI MARTINO, ADRIANA, MD
RESEARCH DIRECTOR
AUTISM CENTER
THE CHILD MIND INSTITUTE
NEW YORK, NY 10022

ESBENSEN, ANNA J, PHD
ASSOCIATE PROFESSOR
DEPARTMENT OF PEDIATRICS
CINCINNATI CHILDREN'S HOSPITAL MEDICAL CENTER
CINCINNATI, OH 45229

EWEN, JOSHUA B, MD
ASSOCIATE PROFESSOR
DEPARTMENT OF NEUROLOGY
KENNEDY KRIEGER INSTITUTE
JOHNS HOPKINS UNIVERSITY
BALTIMORE, MD 21205

FRISTAD, MARY A, PHD
DIRECTOR OF RESEARCH AND DEVELOPMENT
DIRECTOR OF RESEARCH DEVELOPMENT
DEPARTMENT CHILD AND FAMILY PSYCHIATRY
NATIONWIDE CHILDREN'S HOSPITAL BIG LOTS
BEHAVIORAL HEALTH SERVICES
COLUMBUS, OH 43210

GALVAN, ADRIANA, PHD
PROFESSOR
DEPARTMENT OF PSYCHOLOGY
UNIVERSITY OF CALIFORNIA, LOS ANGELES
LOS ANGELES, CA 90095

HERRINGA, RYAN J, MD, PHD
ASSOCIATE PROFESSOR
DEPARTMENT OF PSYCHIATRY
UNIVERSITY OF WISCONSIN, MADISON
MADISON, WI 53719

KNOPIK, VALERIE S, PHD
PROFESSOR
DEPARTMENT OF HUMAN DEVELOPMENT
AND FAMILY STUDIES
PURDUE UNIVERSITY
WEST LAFAYETTE, IN 47907

LERNER, MATTHEW DANIEL, PHD *
ASSOCIATE PROFESSOR
DEPARTMENT OF PSYCHOLOGY
STONY BROOK UNIVERSITY
STONY BROOK, NY 11794

LOO, SANDRA K, PHD
PROFESSOR
DEPARTMENT OF PSYCHIATRY AND
BEHAVIORAL SCIENCES
UNIVERSITY OF CALIFORNIA, LOS ANGELES
LOS ANGELES, CA 90024

MAGANA, SANDRA M, PHD
PROFESSOR
STEVE HICKS SCHOOL OF SOCIAL WORK
THE UNIVERSITY OF TEXAS AT AUSTIN
AUSTIN, TX 78712

MARRUS, NATASHA, PHD, MD *
ASSISTANT PROFESSOR
DEPARTMENT OF PSYCHIATRY
WASHINGTON UNIVERSITY ST. LOUIS
ST. LOUIS, MO 63110

MARTIN, LOREN A, PHD *
PROFESSOR
DEPARTMENT OF CLINICAL PSYCHOLOGY
AZUSA PACIFIC UNIVERSITY
AZUSA, CA 91702

MELHEM, NADINE M, PHD
ASSOCIATE PROFESSOR
DEPARTMENT OF PSYCHIATRY
UNIVERSITY OF PITTSBURGH
PITTSBURGH, PA 15213

MILLER, HAYLIE L, PHD *
ASSISTANT PROFESSOR
SCHOOL OF KINESIOLOGY
UNIVERSITY OF MICHIGAN
ANN ARBOR, MI 48109

MUSSER, ERICA D, PHD *
ASSISTANT PROFESSOR
DEPARTMENT OF PSYCHOLOGY
FLORIDA INTERNATIONAL UNIVERSITY
MIAMI, FL 33199

POSNER, JONATHAN E, MD
PROFESSOR
DEPARTMENT OF PSYCHIATRY AND BEHAVIORAL
SCIENCES
DUKE UNIVERSITY
DURHAM, NC 27708

RIVERA, SUSAN M, PHD
PROFESSOR
DEPARTMENT OF PSYCHOLOGY
UNIVERSITY OF CALIFORNIA, DAVIS
DAVIS, CA 95618

ROBINS, DIANA L, PHD
PROFESSOR
AJ DREXEL AUTISM INSTITUTE
DREXEL UNIVERSITY
PHILADELPHIA, PA 19104

ROOKER, GRIFFIN WESLEY, PHD *
ASSISTANT PROFESSOR
DEPARTMENT OF PSYCHOLOGY
MOUNT ST. MARY'S UNIVERSITY
EMMITSBURG, MD 21727

ROSENBLAU, GABRIELA, PHD *
ASSISTANT PROFESSOR
DEPARTMENT OF PSYCHOLOGICAL AND BRAIN SCIENCES
GEORGE WASHINGTON UNIVERSITY
WASHINGTON, DC 20052

SCHERF, KATHRYN SUZANNE, PHD *
ASSOCIATE PROFESSOR
DEPARTMENT OF PSYCHOLOGY
PENNSYLVANIA STATE UNIVERSITY
UNIVERSITY PARK, PA 16802

SHIC, FREDERICK, PHD
ASSOCIATE PROFESSOR
DEPARTMENT OF PEDIATRICS
UNIVERSITY OF WASHINGTON
SEATTLE, WA 98121

STEPHEN, JULIA MARIE, PHD *
PROFESSOR
DEPARTMENT OF TRANSLATIONAL NEUROSCIENCE
THE MIND RESEARCH NETWORK
UNIVERSITY OF NEW MEXICO
ALBUQUERQUE, NM 87106

STODDARD, JOEL JOHNSON, MD *
ASSOCIATE PROFESSOR
DEPARTMENT OF PSYCHIATRY
UNIVERSITY OF COLORADO
AURORA, CO 80045

SWANSON, MEGHAN RAE, PHD *
ASSISTANT PROFESSOR
DEPARTMENT OF PSYCHOLOGY
THE UNIVERSITY OF TEXAS AT DALLAS
RICHARDSON, TX 75080

THIEMANN-BOURQUE, KATHY, PHD *
ASSOCIATE PROFESSOR
JUNIPER GARDENS CHILDREN'S PROJECT
UNIVERSITY OF KANSAS
KANSAS CITY, KS 66101

TSENG, WAN-LING, PHD *
ASSISTANT PROFESSOR
YALE CHILD STUDY CENTER
YALE UNIVERSITY SCHOOL OF MEDICINE
NEW HAVEN, CT 06519

VALDOVINOS, MARIA G, PHD
PROFESSOR
DEPARTMENT OF PSYCHOLOGY
DRAKE UNIVERSITY
DES MOINES, IA 50311

WHITE, SUSAN WILLIAMS, PHD
PROFESSOR
DEPARTMENT OF PSYCHOLOGY
THE UNIVERSITY OF ALABAMA
TUSCALOOSA, AL 35487

SCIENTIFIC REVIEW OFFICER

SEYMOUR, KAREN ELIZABETH, PHD
SCIENTIFIC REVIEW OFFICER
CENTER FOR SCIENTIFIC REVIEW
NATIONAL INSTITUTE OF HEALTH
BETHESDA, MD 20892

EXTRAMURAL SUPPORT ASSISTANT

SANDERS, MARION
EXTRAMURAL SUPPORT ASSISTANT
CENTER FOR SCIENTIFIC REVIEW
NATIONAL INSTITUTES OF HEALTH
BETHESDA, MD 20892

OTHER REVIEW STAFF

SHAPERO, BENJAMIN GREENBERG, PHD
SCIENTIFIC REVIEW OFFICER
CENTER FOR SCIENTIFIC REVIEW
NATIONAL INSTITUTES OF HEALTH
BETHESDA, MD 20892

* Temporary Member. For grant applications, temporary members may participate in the entire meeting or may review only selected applications as needed.

Consultants are required to absent themselves from the room during the review of any application if their presence would constitute or appear to constitute a conflict of interest.
