# Supplementary material for: Novel Smartphone App and Supportive Accountability for the Treatment of Childhood Disruptive Behavior Problems: Protocol for a Randomized Controlled Trial
Source: JMIR Res Protoc. 2025 Mar 11;14:e67051. doi: 10.2196/67051 (PMC11937717; doi:10.2196/67051)
Supplement: Multimedia Appendix 2 [file resprot_v14i1e67051_app2.pdf]

CONSORT-EHEALTH (V 1.6.1) - Submission/Publication Form

The CONSORT-EHEALTH checklist is intended for authors of randomized trials evaluating web-based and Internet-based applications/interventions, including mobile interventions, electronic games (incl multiplayer games), social media, certain telehealth applications, and other interactive and/or networked electronic applications. Some of the items (e.g. all subitems under item 5 - description of the intervention) may also be applicable for other study designs.

The goal of the CONSORT EHEALTH checklist and guideline is to be
a) a guide for reporting for authors of RCTs,
b) to form a basis for appraisal of an ehealth trial (in terms of validity)

CONSORT-EHEALTH items/subitems are MANDATORY reporting items for studies published in the Journal of Medical Internet Research and other journals / scientific societies endorsing the checklist.

Items numbered 1., 2., 3., 4a., 4b etc are original CONSORT or CONSORT-NPT (non-pharmacologic treatment) items.
Items with Roman numerals (i., ii, iii, iv etc.) are CONSORT-EHEALTH extensions/clarifications.

As the CONSORT-EHEALTH checklist is still considered in a formative stage, we would ask that you also RATE ON A SCALE OF 1-5 how important/useful you feel each item is FOR THE PURPOSE OF THE CHECKLIST and reporting guideline (optional).

Mandatory reporting items are marked with a red *.
In the textboxes, either copy & paste the relevant sections from your manuscript into this form - please include any quotes from your manuscript in QUOTATION MARKS,
or answer directly by providing additional information not in the manuscript, or elaborating on why the item was not relevant for this study.

YOUR ANSWERS WILL BE PUBLISHED AS A SUPPLEMENTARY FILE TO YOUR PUBLICATION IN JMIR AND ARE CONSIDERED PART OF YOUR PUBLICATION (IF ACCEPTED).
Please fill in these questions diligently. Information will not be copyedited, so please use proper spelling and grammar, use correct capitalization, and avoid abbreviations.

DO NOT FORGET TO SAVE AS PDF _AND_ CLICK THE SUBMIT BUTTON SO YOUR ANSWERS ARE IN OUR DATABASE !!!

Citation Suggestion (if you append the pdf as Appendix we suggest to cite this paper in the caption):
Eysenbach G, CONSORT-EHEALTH Group
CONSORT-EHEALTH: Improving and Standardizing Evaluation Reports of Web-based and Mobile Health Interventions
J Med Internet Res 2011;13(4):e126
URL: http://www.jmir.org/2011/4/e126/
doi: 10.2196/jmir.1923
PMID: 22209829

Sign in to Google to save your progress. Learn more

* Indicates required question

Your name *

First Last

Claire Tomlinson

Primary Affiliation (short), City, Country *

University of Toronto, Toronto, Canada

University of Pittsburgh

Your e-mail address *

abc@gmail.com

tomlinsoncs@upmc.edu

Title of your manuscript *

Provide the (draft) title of your manuscript.

A Novel Smartphone-Based Application and Supportive Accountability for the Treatment of Childhood Disruptive Behavior Problems: Protocol for a Randomized Controlled Trial

Name of your App/Software/Intervention *

If there is a short and a long/alternate name, write the short name first and add the long name in brackets.

UseIt!

Evaluated Version (if any)

e.g. "V1", "Release 2017-03-01", "Version 2.0.27913"

Your answer

Language(s) *

What language is the intervention/app in? If multiple languages are available, separate by comma (e.g. "English, French")

English

URL of your Intervention Website or App

e.g. a direct link to the mobile app on app in appstore (itunes, Google Play), or URL of the website. If the intervention is a DVD or hardware, you can also link to an Amazon page.

https://apps.apple.com/us/app/useit/id1633301592

URL of an image/screenshot (optional)

Your answer

Accessibility *

Can an enduser access the intervention presently?

- ( ) access is free and open
- (•) access only for special usergroups, not open
- ( ) access is open to everyone, but requires payment/subscription/in-app purchases
- ( ) app/intervention no longer accessible
- ( ) Other:

Primary Medical Indication/Disease/Condition *

e.g. "Stress", "Diabetes", or define the target group in brackets after the condition, e.g. "Autism (Parents of children with)", "Alzheimers (Informal Caregivers of)"

Behavior Problems in Children

Primary Outcomes measured in trial *

comma-separated list of primary outcomes reported in the trial

Parent knowledge of parent management train

Secondary/other outcomes

Are there any other outcomes the intervention is expected to affect?

use of evidence-based parenting strategies, behavior problems in children, parent mental health concerns

Recommended "Dose" *

What do the instructions for users say on how often the app should be used?

- (selected) Approximately Daily
- Approximately Weekly
- Approximately Monthly
- Approximately Yearly
- "as needed"
- Other:

Approx. Percentage of Users (starters) still using the app as recommended after 3 months *

- ○ unknown / not evaluated
- ○ 0-10%
- ○ 11-20%
- ◉ 21-30%
- ○ 31-40%
- ○ 41-50%
- ○ 51-60%
- ○ 61-70%
- ○ 71%-80%
- ○ 81-90%
- ○ 91-100%
- ○ Other:

Overall, was the app/intervention effective? *

- ( ) yes: all primary outcomes were significantly better in intervention group vs control
- ( ) partly: SOME primary outcomes were significantly better in intervention group vs control
- ( ) no statistically significant difference between control and intervention
- ( ) potentially harmful: control was significantly better than intervention in one or more outcomes
- ( ) inconclusive: more research is needed
- (•) Other: data not yet fully collected

Article Preparation Status/Stage *

At which stage in your article preparation are you currently (at the time you fill in this form)

- (•) not submitted yet - in early draft status
- ( ) not submitted yet - in late draft status, just before submission
- ( ) submitted to a journal but not reviewed yet
- ( ) submitted to a journal and after receiving initial reviewer comments
- ( ) submitted to a journal and accepted, but not published yet
- ( ) published
- ( ) Other:

Journal *

If you already know where you will submit this paper (or if it is already submitted), please provide the journal name (if it is not JMIR, provide the journal name under "other")

- (x) not submitted yet / unclear where I will submit this
- ( ) Journal of Medical Internet Research (JMIR)
- ( ) JMIR mHealth and UHealth
- ( ) JMIR Serious Games
- ( ) JMIR Mental Health
- ( ) JMIR Public Health
- ( ) JMIR Formative Research
- ( ) Other JMIR sister journal
- ( ) Other:

Is this a full powered effectiveness trial or a pilot/feasibility trial? *

- ( ) Pilot/feasibility
- (x) Fully powered

Manuscript tracking number *

If this is a JMIR submission, please provide the manuscript tracking number under "other" (The ms tracking number can be found in the submission acknowledgement email, or when you login as author in JMIR. If the paper is already published in JMIR, then the ms tracking number is the four-digit number at the end of the DOI, to be found at the bottom of each published article in JMIR)

- [x] no ms number (yet) / not (yet) submitted to / published in JMIR
- [ ] Other:

TITLE AND ABSTRACT

1a) TITLE: Identification as a randomized trial in the title

1a) Does your paper address CONSORT item 1a? *

I.e does the title contain the phrase "Randomized Controlled Trial"? (if not, explain the reason under "other")

- [x] yes
- [ ] Other:

1a-i) Identify the mode of delivery in the title

Identify the mode of delivery. Preferably use “web-based” and/or “mobile” and/or “electronic game” in the title. Avoid ambiguous terms like “online”, “virtual”, “interactive”. Use “Internet-based” only if Intervention includes non-web-based Internet components (e.g. email), use “computer-based” or “electronic” only if offline products are used. Use “virtual” only in the context of “virtual reality” (3-D worlds). Use “online” only in the context of “online support groups”. Complement or substitute product names with broader terms for the class of products (such as “mobile” or “smart phone” instead of “iphone”), especially if the application runs on different platforms.

	1	2	3	4	5	
subitem not at all important	○	○	○	○	◉	essential

Clear selection

Does your paper address subitem 1a-i? *

Copy and paste relevant sections from manuscript title (include quotes in quotation marks "like this" to indicate direct quotes from your manuscript), or elaborate on this item by providing additional information not in the ms, or briefly explain why the item is not applicable/relevant for your study

A Novel Smartphone-Based Application and Supportive Accountability for the Treatment of Childhood Disruptive Behavior Problems: Protocol for a Randomized Controlled Trial

1a-ii) Non-web-based components or important co-interventions in title

Mention non-web-based components or important co-interventions in title, if any (e.g., “with telephone support”).

	1	2	3	4	5	
subitem not at all important	○	○	○	○	◉	essential

Clear selection

Does your paper address subitem 1a-ii?

Copy and paste relevant sections from manuscript title (include quotes in quotation marks "like this" to indicate direct quotes from your manuscript), or elaborate on this item by providing additional information not in the ms, or briefly explain why the item is not applicable/relevant for your study

"A Novel Smartphone-Based Application and Supportive Accountability for the Treatment of Childhood Disruptive Behavior Problems: Protocol for a Randomized Controlled Trial"

1a-iii) Primary condition or target group in the title

Mention primary condition or target group in the title, if any (e.g., “for children with Type I Diabetes”) Example: A Web-based and Mobile Intervention with Telephone Support for Children with Type I Diabetes: Randomized Controlled Trial

	1	2	3	4	5	
subitem not at all important	○	○	○	○	◉	essential

Clear selection

Does your paper address subitem 1a-iii? *

Copy and paste relevant sections from manuscript title (include quotes in quotation marks "like this" to indicate direct quotes from your manuscript), or elaborate on this item by providing additional information not in the ms, or briefly explain why the item is not applicable/relevant for your study

"A Novel Smartphone-Based Application and Supportive Accountability for the Treatment of Childhood Disruptive Behavior Problems: Protocol for a Randomized Controlled Trial"

1b) ABSTRACT: Structured summary of trial design, methods, results, and conclusions

NPT extension: Description of experimental treatment, comparator, care providers, centers, and blinding status.

1b-i) Key features/functionalities/components of the intervention and comparator in the METHODS section of the ABSTRACT

Mention key features/functionalities/components of the intervention and comparator in the abstract. If possible, also mention theories and principles used for designing the site. Keep in mind the needs of systematic reviewers and indexers by including important synonyms. (Note: Only report in the abstract what the main paper is reporting. If this information is missing from the main body of text, consider adding it)

	1	2	3	4	5	
subitem not at all important	○	○	○	◉	○	essential

Clear selection

Does your paper address subitem 1b-i? *

Copy and paste relevant sections from the manuscript abstract (include quotes in quotation marks "like this" to indicate direct quotes from your manuscript), or elaborate on this item by providing additional information not in the ms, or briefly explain why the item is not applicable/relevant for your study

"Methods: A nationwide sample of parents of children ages 5 to 8 years with disruptive behaviors (N = 324 dyads) will be randomly assigned to the standalone app (UseIt!; n = 108), the coach-assisted app (UseIt! plus supportive accountability; n = 108), or the control app (mindfullness app; n = 108). The UseIt! app provides parents with tools and troubleshooting to address disruptive behaviors, along with a behavior diary to track behaviors and strategies over time. The coach-assisted condition includes a bachelor's level paraprofessional who provides weekly phone calls to promote engagement with the app. The control condition is comprised of a mindfulness app. The outcome assessments (post-treatment and 6-month follow-up) include measures of app usage, parenting knowledge (e.g., knowledge of parent management training and cognitive behavioral therapy skills) and strategies (use of evidence-based parenting strategies), symptom reduction (e.g., behavior problems), and parental mental health (e.g., anxiety, stress, depression). We hypothesize that both intervention conditionswill show greater parental knowledge and use of skills along with greater symptom reduction relative to the control condition. Further, we hypothesize that those assigned to the coach assisted condition will report greater knowledge, skill use, and symptom reduction compared to the standalone app. We will use intent to treat analyses to regress outcomes on study conditions to evaluate for differences across conditions."

1b-ii) Level of human involvement in the METHODS section of the ABSTRACT

Clarify the level of human involvement in the abstract, e.g., use phrases like “fully automated” vs. “therapist/nurse/care provider/physician-assisted” (mention number and expertise of providers involved, if any). (Note: Only report in the abstract what the main paper is reporting. If this information is missing from the main body of text, consider adding it)

	1	2	3	4	5	
subitem not at all important	○	○	○	◉	○	essential

Clear selection

Does your paper address subitem 1b-ii?

Copy and paste relevant sections from the manuscript abstract (include quotes in quotation marks "like this" to indicate direct quotes from your manuscript), or elaborate on this item by providing additional information not in the ms, or briefly explain why the item is not applicable/relevant for your study

"Methods: A nationwide sample of parents of children ages 5 to 8 years with disruptive behaviors (N = 324 dyads) will be randomly assigned to the standalone app (UseIt!; n = 108), the coach-assisted app (UseIt! plus supportive accountability; n = 108), or the control app (mindfullness app; n = 108). The UseIt! app provides parents with tools and troubleshooting to address disruptive behaviors, along with a behavior diary to track behaviors and strategies over time. The coach-assisted condition includes a bachelor's level paraprofessional who provides weekly phone calls to promote engagement with the app. The control condition is comprised of a mindfulness app. The outcome assessments (post-treatment and 6-month follow-up) include measures of app usage, parenting knowledge (e.g., knowledge of parent management training and cognitive behavioral therapy skills) and strategies (use of evidence-based parenting strategies), symptom reduction (e.g., behavior problems), and parental mental health (e.g., anxiety, stress, depression). We hypothesize that both intervention conditionswill show greater parental knowledge and use of skills along with greater symptom reduction relative to the control condition. Further, we hypothesize that those assigned to the coach assisted condition will report greater knowledge, skill use, and symptom reduction compared to the standalone app. We will use intent to treat analyses to regress outcomes on study conditions to evaluate for differences across conditions."

1b-iii) Open vs. closed, web-based (self-assessment) vs. face-to-face assessments in the METHODS section of the ABSTRACT

Mention how participants were recruited (online vs. offline), e.g., from an open access website or from a clinic or a closed online user group (closed usergroup trial), and clarify if this was a purely web-based trial, or there were face-to-face components (as part of the intervention or for assessment). Clearly say if outcomes were self-assessed through questionnaires (as common in web-based trials). Note: In traditional offline trials, an open trial (open-label trial) is a type of clinical trial in which both the researchers and participants know which treatment is being administered. To avoid confusion, use “blinded” or “unblinded” to indicated the level of blinding instead of “open”, as “open” in web-based trials usually refers to “open access” (i.e. participants can self-enrol). (Note: Only report in the abstract what the main paper is reporting. If this information is missing from the main body of text, consider adding it)

	1	2	3	4	5	
subitem not at all important	○	○	○	●	○	essential

Clear selection

Does your paper address subitem 1b-iii?

Copy and paste relevant sections from the manuscript abstract (include quotes in quotation marks "like this" to indicate direct quotes from your manuscript), or elaborate on this item by providing additional information not in the ms, or briefly explain why the item is not applicable/relevant for your study

"Methods: A nationwide sample of parents of children ages 5 to 8 years with disruptive behaviors (N = 324 dyads) will be randomly assigned to the standalone app (UseIt!; n = 108), the coach-assisted app (UseIt! plus supportive accountability; n = 108), or the control app (mindfullness app; n = 108). The UseIt! app provides parents with tools and troubleshooting to address disruptive behaviors, along with a behavior diary to track behaviors and strategies over time. The coach-assisted condition includes a bachelor's level paraprofessional who provides weekly phone calls to promote engagement with the app. The control condition is comprised of a mindfulness app. The online, self-assessed outcome measures (post-treatment and 6-month follow-up) include measures of app usage, parenting knowledge (e.g., knowledge of parent management training and cognitive behavioral therapy skills) and strategies (use of evidence-based parenting strategies), symptom reduction (e.g., behavior problems), and parental mental health (e.g., anxiety, stress, depression). We hypothesize that both intervention conditionswill show greater parental knowledge and use of skills along with greater symptom reduction relative to the control condition. Further, we hypothesize that those assigned to the coach assisted condition will report greater knowledge, skill use, and symptom reduction compared to the standalone app. We will use intent to treat analyses to regress outcomes on study conditions to evaluate for differences across conditions."

1b-iv) RESULTS section in abstract must contain use data

Report number of participants enrolled/assessed in each group, the use/uptake of the intervention (e.g., attrition/adherence metrics, use over time, number of logins etc.), in addition to primary/secondary outcomes. (Note: Only report in the abstract what the main paper is reporting. If this information is missing from the main body of text, consider adding it)

	1	2	3	4	5	
subitem not at all important	○	○	○	●	○	essential

Clear selection

Does your paper address subitem 1b-iv?

Copy and paste relevant sections from the manuscript abstract (include quotes in quotation marks "like this" to indicate direct quotes from your manuscript), or elaborate on this item by providing additional information not in the ms, or briefly explain why the item is not applicable/relevant for your study

"Results: Recruitment of study participants began in December of 2022 and is ongoing. We have recruited just over half of our intended sample of 324 parent-child dyads (n=224) as of December, 2024. These dyads were randomly allocated to each of the intervention conditions, with 71 assigned to the coach-assisted condition, 72 assigned to the standalone app, and 71 assigned to the control app condition. Data collection is projected to be completed by late 2026."
The current manuscript reports only the protocol for the forthcoming RCT, so the results are truncated.

1b-v) CONCLUSIONS/DISCUSSION in abstract for negative trials

Conclusions/Discussions in abstract for negative trials: Discuss the primary outcome - if the trial is negative (primary outcome not changed), and the intervention was not used, discuss whether negative results are attributable to lack of uptake and discuss reasons. (Note: Only report in the abstract what the main paper is reporting. If this information is missing from the main body of text, consider adding it)

	1	2	3	4	5	
subitem not at all important	○	○	○	◉	○	essential

Clear selection

Does your paper address subitem 1b-v?

Copy and paste relevant sections from the manuscript abstract (include quotes in quotation marks "like this" to indicate direct quotes from your manuscript), or elaborate on this item by providing additional information not in the ms, or briefly explain why the item is not applicable/relevant for your study

"Conclusions: The current study aims to address a gap in the literature regarding the feasibility, effectiveness, and utility of a smartphone-based application that includes a coach-assisted arm. Digital therapeutics have the potential to enhance the reach and scalability of skills-based psychosocial interventions. Findings from the current study will advance scientific knowledge and have implications for clinical practice."

INTRODUCTION

2a) In INTRODUCTION: Scientific background and explanation of rationale

2a-i) Problem and the type of system/solution

Describe the problem and the type of system/solution that is object of the study: intended as stand-alone intervention vs. incorporated in broader health care program? Intended for a particular patient population? Goals of the intervention, e.g., being more cost-effective to other interventions, replace or complement other solutions? (Note: Details about the intervention are provided in “Methods” under 5)

	1	2	3	4	5	
subitem not at all important	○	○	○	○	◉	essential

Clear selection

Does your paper address subitem 2a-i? *

Copy and paste relevant sections from the manuscript (include quotes in quotation marks "like this" to indicate direct quotes from your manuscript), or elaborate on this item by providing additional information not in the ms, or briefly explain why the item is not applicable/relevant for your study

"DBDs are typically treated with psychosocial evidence-based therapies [4] that include parent-management training (PMT) skills (e.g., praise, rewards, consequences, time-outs) and cognitive-behavioral therapy (CBT) skills (e.g., problem-solving, emotion labeling) [5]. Meta-analyses point to the substantial effectiveness of these interventions at reducing symptoms and maintaining treatment gains over time [6,7]. Despite the effectiveness of EBTs, many families do not have access to these services, and often stop attending or fail to practice new skills between sessions. Barriers to access include local availability of services, transportation, cost, and perceived stigma. Barriers to non-completion include poor motivation and low engagement, along with competing demands for time, transportation problems, and co-payment costs [8]."

"Recent advances in technology, in particular mobile health (mHealth) systems, have the potential to overcome these barriers, and promote better data collection for researchers [9]. mHealth technologies, including smartphones, create an opportunity to develop personalized interventions that are delivered to families in their day-to-day settings [10]. A smartphone based mHealth system has numerous potential advantages for improving access to, and engagement in, EBTs for childhood behavior problems. Such applications can deliver content to improve understanding of skills, can provide opportunities for learning through skills practice, and can give feedback to families regarding areas for improvement [11,12]."

2a-ii) Scientific background, rationale: What is known about the (type of) system

Scientific background, rationale: What is known about the (type of) system that is the object of the study (be sure to discuss the use of similar systems for other conditions/diagnoses, if appropiate), motivation for the study, i.e. what are the reasons for and what is the context for this specific study, from which stakeholder viewpoint is the study performed, potential impact of findings [2]. Briefly justify the choice of the comparator.

	1	2	3	4	5	
subitem not at all important	○	○	○	○	◉	essential

Clear selection

Does your paper address subitem 2a-ii? *

Copy and paste relevant sections from the manuscript (include quotes in quotation marks "like this" to indicate direct quotes from your manuscript), or elaborate on this item by providing additional information not in the ms, or briefly explain why the item is not applicable/relevant for your study

"Rigorous evaluations of mHealth interventions targeting child behavioral concerns are in their nascent stage. A 2024 systematic review of mHealth interventions targeting behavioral problems in youth indicated a wide range of effect sizes, from small to large, across the 11 studies reviewed. Most sample sizes were small (i.e., less than 100), and the few larger studies did not focus on clinical outcomes (e.g., satisfaction, acceptability and app usage, without behavioral outcomes) [2]. Other recent studies reflect similar findings (i.e., improvements in child and parental symptoms, with a wide range of effect sizes), but suffer from similar limitations (i.e., small sample sizes, outcome measures focused on satisfaction or acceptability of apps rather than empirically based measures of symptom improvement) [3–6]."

"The Role of the Coach in mHealth
There is growing evidence in the field of digital therapeutics that some degree of human interaction is important to sustain app usage and achieve meaningful outcomes [17–19]. Although various models have been proposed, the “Coaching” model affords many of the benefits of human interaction (e.g., support, accountability) at a level of service that remains highly scalable [17]. Mohr’s Supportive Accountability (SA) Model, which is flexible and can be tailored to clinical conditions and service users, details that the intervention is supported by a coach who provides a social presence and accountability to boost motivation and engagement. Effectiveness studies have shown that various paraprofessionals can successfully be trained as coaches for a wide range of interventions and clinical populations [17]."

2b) In INTRODUCTION: Specific objectives or hypotheses

Does your paper address CONSORT subitem 2b? *

Copy and paste relevant sections from the manuscript (include quotes in quotation marks "like this" to indicate direct quotes from your manuscript), or elaborate on this item by providing additional information not in the ms, or briefly explain why the item is not applicable/relevant for your study

"Primary Aim
We aim to evaluate the effectiveness of the UseIt! mHealth system as both a standalone (n = 108) and coach-assisted (n = 108) intervention compared to a control app condition (n = 108). We expect that 1) the two UseIt! intervention conditions will score higher on parenting knowledge (primary outcome) and show greater post-treatment reductions in disruptive behavior symptoms (secondary outcome) compared to the control condition, and 2) the coach-assisted UseIt! condition will score higher on parenting knowledge and show greater post-treatment reductions compared to the standalone UseIt! condition."
"Secondary Aims
We will also test mechanisms of therapeutic change. In particular, we will test whether gains in knowledge of parenting skills are associated with reductions in disruptive behavior symptoms. Lastly, we aim to evaluate the effectiveness of the components of the UseIt! mHealth system. We will compare app usage across the standalone and coach-assisted conditions and test whether the app usage indices are associated with target engagement, knowledge of parenting skills, and symptom reduction at post-treatment. We expect that families who use the app more often will have higher skill acquisition/utilization scores at post-treatment ("dose" effects) though we do not have specific hypotheses regarding individual app features."

METHODS

3a) Description of trial design (such as parallel, factorial) including allocation ratio

Does your paper address CONSORT subitem 3a? *

Copy and paste relevant sections from the manuscript (include quotes in quotation marks "like this" to indicate direct quotes from your manuscript), or elaborate on this item by providing additional information not in the ms, or briefly explain why the item is not applicable/relevant for your study

"The study is a randomized controlled trial with three conditions. Parents of children aged 5 to 8 (n=324) are randomly assigned to one of three conditions: a standalone UseIt! app condition, a UseIt! app + Coach condition, and a control app condition (Smiling Mind app). Data will be collected via online surveys at baseline, post-treatment (four months after baseline), and at 6-month follow-up. Randomization takes place after the baseline is completed."

3b) Important changes to methods after trial commencement (such as eligibility criteria), with reasons

Does your paper address CONSORT subitem 3b? *

Copy and paste relevant sections from the manuscript (include quotes in quotation marks "like this" to indicate direct quotes from your manuscript), or elaborate on this item by providing additional information not in the ms, or briefly explain why the item is not applicable/relevant for your study

No changes made.

Your answer must have a minimum of 25 characters.

3b-i) Bug fixes, Downtimes, Content Changes

Bug fixes, Downtimes, Content Changes: ehealth systems are often dynamic systems. A description of changes to methods therefore also includes important changes made on the intervention or comparator during the trial (e.g., major bug fixes or changes in the functionality or content) (5-iii) and other “unexpected events” that may have influenced study design such as staff changes, system failures/downtimes, etc. [2].

	1	2	3	4	5	
subitem not at all important	○	○	○	○	◉	essential

Clear selection

Does your paper address subitem 3b-i?

Copy and paste relevant sections from the manuscript (include quotes in quotation marks "like this" to indicate direct quotes from your manuscript), or elaborate on this item by providing additional information not in the ms, or briefly explain why the item is not applicable/relevant for your study

The current manuscript is a protocol, and thus changes to the system are not complete at this point.

4a) Eligibility criteria for participants

Does your paper address CONSORT subitem 4a? *

Copy and paste relevant sections from the manuscript (include quotes in quotation marks "like this" to indicate direct quotes from your manuscript), or elaborate on this item by providing additional information not in the ms, or briefly explain why the item is not applicable/relevant for your study

"Participant Eligibility

Inclusion criteria for the current study requires that parents/guardians have 1) a child between the ages of 5 and 8, 2) that child must be above the 90th percentile for Oppositional Defiant Disorder and/or Conduct Disorder on the Vanderbilt Assessment Scale, 3) the child must be in residence with the parent/guardian for at least 80% of the time, 4) parent/guardian must consent to study participation, and 5) parent/guardian must have a smartphone device with daily internet access. Exclusion criteria includes if the child 1) has a known preexisting behavioral or mental health diagnosis requiring alternative treatment (e.g., bipolar disorder, major depression, pervasive developmental disorder) or 2) is currently in treatment for childhood disruptive behavior."

4a-i) Computer / Internet literacy

Computer / Internet literacy is often an implicit “de facto” eligibility criterion - this should be explicitly clarified.

	1	2	3	4	5	
subitem not at all important	○	○	○	◉	○	essential

Clear selection

Does your paper address subitem 4a-i?

Copy and paste relevant sections from the manuscript (include quotes in quotation marks "like this" to indicate direct quotes from your manuscript), or elaborate on this item by providing additional information not in the ms, or briefly explain why the item is not applicable/relevant for your study

We assess technological literacy as a baseline measure, described here, below. It was not part of the eligibility criteria.

"Technological Literacy
The Technological Self-Assessment Scale (TSAT [38]) is a 13-item parent report screen for technological ability. The measure was created for the current study by the research team to provide an indicator of parent knowledge and experience with their computers and phones. For example, items ask parents if they know how to search for information on the internet, if they have ever downloaded an app, and if they have social media accounts. Items are scored Yes/No, and scores range from 0-13."

4a-ii) Open vs. closed, web-based vs. face-to-face assessments:

Open vs. closed, web-based vs. face-to-face assessments: Mention how participants were recruited (online vs. offline), e.g., from an open access website or from a clinic, and clarify if this was a purely web-based trial, or there were face-to-face components (as part of the intervention or for assessment), i.e., to what degree got the study team to know the participant. In online-only trials, clarify if participants were quasi-anonymous and whether having multiple identities was possible or whether technical or logistical measures (e.g., cookies, email confirmation, phone calls) were used to detect/prevent these.

	1	2	3	4	5	
subitem not at all important	○	○	○	○	◉	essential

Clear selection

Does your paper address subitem 4a-ii? *

Copy and paste relevant sections from the manuscript (include quotes in quotation marks "like this" to indicate direct quotes from your manuscript), or elaborate on this item by providing additional information not in the ms, or briefly explain why the item is not applicable/relevant for your study

"Study Procedures and Randomization
Participants provide their contact information to the Pitt + Me or BuildClinical systems after accessing the study advertisement. Trained research assistants then contact families and conduct the initial screening to determine study eligibility. If determined eligible, a future call is scheduled to obtain consent from the parent, and assent from the child to participate. After consent, parents are provided a Qualtrics link to complete the initial baseline assessment. After completion of the assessment, families are randomly assigned to Group 1 (standalone UseIt! app; n = 108), Group 2 (UseIt! app + coach; n = 108), or Group 3 (control app condition; n = 108). We use stratified randomization to ensure that the groups are equivalent on key clinical features (screening severity and referral source (i.e., Pitt + Me or BuildClinical). Parents assigned to the Coach condition will be walked through the initial setup and login process, along with a brief training on how to use the applications over the phone. Families can be set up and trained in approximately 30 minutes. If parents cannot be reached after three weeks, instructions are sent via email. Parents assigned to the standalone UseIt! app condition are sent a tutorial video with the same information. Parents use the app condition assigned for four months before the administration of the post-treatment assessment, via Qualtrics. Six months following the post-treatment assessment, parents again are prompted to complete the 6-month follow-up assessment via Qualtrics."

4a-iii) Information giving during recruitment

Information given during recruitment. Specify how participants were briefed for recruitment and in the informed consent procedures (e.g., publish the informed consent documentation as appendix, see also item X26), as this information may have an effect on user self-selection, user expectation and may also bias results.

	1	2	3	4	5	
subitem not at all important	○	○	○	○	◉	essential

Clear selection

Does your paper address subitem 4a-iii?

Copy and paste relevant sections from the manuscript (include quotes in quotation marks "like this" to indicate direct quotes from your manuscript), or elaborate on this item by providing additional information not in the ms, or briefly explain why the item is not applicable/relevant for your study

"Study Procedures and Randomization
Participants provide their contact information to the Pitt + Me or BuildClinical systems after accessing the study advertisement. Trained research assistants then contact families and conduct the initial screening to determine study eligibility. If determined eligible, a future call is scheduled to obtain consent from the parent, and assent from the child to participate. After consent, parents are provided a Qualtrics link to complete the initial baseline assessment. After completion of the assessment, families are randomly assigned to Group 1 (standalone UseIt! app; n = 108), Group 2 (UseIt! app + coach; n = 108), or Group 3 (control app condition; n = 108). We use stratified randomization to ensure that the groups are equivalent on key clinical features (screening severity and referral source (i.e., Pitt + Me or BuildClinical). Parents assigned to the Coach condition will be walked through the initial setup and login process, along with a brief training on how to use the applications over the phone. Families can be set up and trained in approximately 30 minutes. If parents cannot be reached after three weeks, instructions are sent via email. Parents assigned to the standalone UseIt! app condition are sent a tutorial video with the same information. Parents use the app condition assigned for four months before the administration of the post-treatment assessment, via Qualtrics. Six months following the post-treatment assessment, parents again are prompted to complete the 6-month follow-up assessment via Qualtrics."

4b) Settings and locations where the data were collected

Does your paper address CONSORT subitem 4b? *

Copy and paste relevant sections from the manuscript (include quotes in quotation marks "like this" to indicate direct quotes from your manuscript), or elaborate on this item by providing additional information not in the ms, or briefly explain why the item is not applicable/relevant for your study

All data was collected remotely, described in study procedures and randomization, below:
"Study Procedures and Randomization
Participants provide their contact information to the Pitt + Me or BuildClinical systems after accessing the study advertisement. Trained research assistants then contact families and conduct the initial screening to determine study eligibility. If determined eligible, a future call is scheduled to obtain consent from the parent, and assent from the child to participate. After consent, parents are provided a Qualtrics link to complete the initial baseline assessment. After completion of the assessment, families are randomly assigned to Group 1 (standalone UseIt! app; n = 108), Group 2 (UseIt! app + coach; n = 108), or Group 3 (control app condition; n = 108). We use stratified randomization to ensure that the groups are equivalent on key clinical features (screening severity and referral source (i.e., Pitt + Me or BuildClinical). Parents assigned to the Coach condition will be walked through the initial setup and login process, along with a brief training on how to use the applications over the phone. Families can be set up and trained in approximately 30 minutes. If parents cannot be reached after three weeks, instructions are sent via email. Parents assigned to the standalone UseIt! app condition are sent a tutorial video with the same information. Parents use the app condition assigned for four months before the administration of the post-treatment assessment, via Qualtrics. Six months following the post-treatment assessment, parents again are prompted to complete the 6-month follow-up assessment via Qualtrics."

4b-i) Report if outcomes were (self-)assessed through online questionnaires

Clearly report if outcomes were (self-)assessed through online questionnaires (as common in web-based trials) or otherwise.

	1	2	3	4	5	
subitem not at all important	○	○	○	○	◉	essential

Clear selection

Does your paper address subitem 4b-i? *

Copy and paste relevant sections from the manuscript (include quotes in quotation marks "like this" to indicate direct quotes from your manuscript), or elaborate on this item by providing additional information not in the ms, or briefly explain why the item is not applicable/relevant for your study

"Study Procedures and Randomization
Participants provide their contact information to the Pitt + Me or BuildClinical systems after accessing the study advertisement. Trained research assistants then contact families and conduct the initial screening to determine study eligibility. If determined eligible, a future call is scheduled to obtain consent from the parent, and assent from the child to participate. After consent, parents are provided a Qualtrics link to complete the initial baseline assessment. After completion of the assessment, families are randomly assigned to Group 1 (standalone UseIt! app; n = 108), Group 2 (UseIt! app + coach; n = 108), or Group 3 (control app condition; n = 108). We use stratified randomization to ensure that the groups are equivalent on key clinical features (screening severity and referral source (i.e., Pitt + Me or BuildClinical). Parents assigned to the Coach condition will be walked through the initial setup and login process, along with a brief training on how to use the applications over the phone. Families can be set up and trained in approximately 30 minutes. If parents cannot be reached after three weeks, instructions are sent via email. Parents assigned to the standalone UseIt! app condition are sent a tutorial video with the same information. Parents use the app condition assigned for four months before the administration of the post-treatment assessment, via Qualtrics. Six months following the post-treatment assessment, parents again are prompted to complete the 6-month follow-up assessment via Qualtrics."

4b-ii) Report how institutional affiliations are displayed

Report how institutional affiliations are displayed to potential participants [on ehealth media], as affiliations with prestigious hospitals or universities may affect volunteer rates, use, and reactions with regards to an intervention.(Not a required item – describe only if this may bias results)

	1	2	3	4	5	
subitem not at all important	○	○	○	◉	○	essential

Clear selection

Does your paper address subitem 4b-ii?

Copy and paste relevant sections from the manuscript (include quotes in quotation marks "like this" to indicate direct quotes from your manuscript), or elaborate on this item by providing additional information not in the ms, or briefly explain why the item is not applicable/relevant for your study

"Recruitment
We will use a two-pronged recruitment strategy to maximize enrollment. The two recruitment avenues will be: 1) the Clinical and Translational Science Institute (CTSI) patient registry (Pitt+Me®) at the University of Pittsburgh, and 2) BuildClinical. The CTSI patient registry (Pitt+Me®) is an institutional research participant registry that uses enhanced study descriptions and social media to engage the community in research. BuildClinical is a clinical trial recruiting system that helps investigators recruit participants for clinical trials more efficiently. Using study-specific digital advertisements displayed on search engines, health websites, and social media platforms, BuildClinical generates participant referrals. BuildClinical also provides tools to streamline the recruitment and prescreening process. The platform stores information in a HIPAA compliant manner and allows for remote enrollment."

5) The interventions for each group with sufficient details to allow replication, including how and when they were actually administered

5-i) Mention names, credential, affiliations of the developers, sponsors, and owners

Mention names, credential, affiliations of the developers, sponsors, and owners [6] (if authors/evaluators are owners or developer of the software, this needs to be declared in a "Conflict of interest" section or mentioned elsewhere in the manuscript).

	1	2	3	4	5	
subitem not at all important	○	○	○	○	◉	essential

Clear selection

Does your paper address subitem 5-i?

Copy and paste relevant sections from the manuscript (include quotes in quotation marks "like this" to indicate direct quotes from your manuscript), or elaborate on this item by providing additional information not in the ms, or briefly explain why the item is not applicable/relevant for your study

"UseIt! Smartphone Application
The UseIt! system includes a cross-platform mHealth app that runs on both iOS and Android devices. The app is securely connected to a portal where app feature usage is stored. The portal was designed for the research team to track and monitor usage of the app by parents. The app contains six features: (1) a troubleshooting guide that provides detailed skill recommendations for problem situations, (2) a behavior diary for tracking behaviors and skills used each day, (3) a digital library that provides definitions and instructions for each skill, (4) a point counter for parents to award points to their children, (5) a skills-alarm for reminding parents to practice the various skills, and (6) a timer for use with parenting skills (e.g., time-outs, managing screen time, routines). Users can examine diary entries, view points awarded, and set the skills-alarm through the app. See Figure 1 for a screenshot of the App home page. [Insert Figure 1 here]
Troubleshooting Guide
The UseIt! troubleshooting guide contains information to help parents effectively respond [illegible]

to problem-behaviors. Parents are presented with a list of potential negative behaviors (e.g. bullied or fought). After selecting a behavior, appropriate skill options (e.g. time-out) are displayed with tips to effectively apply each skill. Once a skill has been used and the behavior has stopped, parents are reminded to praise their child for positive behaviors. See Figure 2. [Insert Figure 2 here]
Behavior Diary
The behavior diary cues participants (via a notification) to complete a series of questions about behaviors and PMT/CBT skills used each day. The results are displayed graphically and can be reviewed by the user to track progress over time. This allows the user to keep track of what skills the family has tried for different behaviors (both positive and negative child behaviors) and which ones have been helpful in various contexts. See Figure 3. [Insert Figure 3 here]
Digital Library
The UseIt! digital library provides detailed information about using strategies for positive and negative child behavior. Each skill is defined and presented with tips for how and when to effectively use each skill. The digital library contains more information than the troubleshooting guide and is designed as an information source for reviewing PMT/CBT topics. See Figure 4. [Insert Figure 4 here]
Point Counter
Treatment for disruptive behavior disorders typically includes prizes for treatment adherence, positive behaviors, and skill use. Parents can award points to children for target behaviors (e.g. cleaning dishes) and skill utilization. The UseIt! point counter features an on-screen button ("Give your child a point") which parents press to reward their child with a point. The feature functions as a digital rewards program that parents can use to keep track of points and reward their children. See Figure 1 for a view of the point counter window.
Skills Alarm
Skills alarms can be set at any time via the app. Users can set dates and times for notifications to activate. These notifications remind parents and children to practice specific skills throughout the week (e.g. "remember to praise your child"). Parents are able to view a list of active and inactive alarms. See Figure 5. [Insert Figure 5 here]
Timer
Timers can be set for use with a variety of skills (e.g., time-outs, screen time management, routines) to promote use of the skills."

5-ii) Describe the history/development process

Describe the history/development process of the application and previous formative evaluations (e.g., focus groups, usability testing), as these will have an impact on adoption/use rates and help with interpreting results.

	1	2	3	4	5	
subitem not at all important	○	○	○	○	◉	essential

Clear selection

Does your paper address subitem 5-ii?

Copy and paste relevant sections from the manuscript (include quotes in quotation marks "like this" to indicate direct quotes from your manuscript), or elaborate on this item by providing additional information not in the ms, or briefly explain why the item is not applicable/relevant for your study

"Prior Pilot Trials of UseIt! mHealth System
The UseIt! app combines aspects of Parent Management Training (PMT) and Cognitive Behavioral Therapy (CBT) to provide parents with evidence-based skills to decrease disruptive behaviors in their children. To date, we have completed two pilot RCTs to test preliminary target engagement and effectiveness of the UseIt! mHealth system The first trial tested the UseIt! mHealth system as a standalone intervention (N = 34). Parent-child dyads enrolled in the study and were randomly assigned to either the UseIt! mHealth app condition (n = 17) or ta waitlist condition (n = 17). Overall, results supported the feasibility of the intervention but attrition was high in the waitlist control group. This informed our decision to have an active control group rather than a waitlist control group in the current study. The second pilot trial tested the UseIt! mHealth system as an adjunct to therapy in community settings (N=39 parent-child dyads). Though treatment targets moved in the expected direction, high clinician turnover in the community settingslimited the sustainability and scalability of this approach. This informed our decision to include a bachelor’s level paraprofessional “coach”rather than a clinician in the current study."

5-iii) Revisions and updating

Revisions and updating. Clearly mention the date and/or version number of the application/intervention (and comparator, if applicable) evaluated, or describe whether the intervention underwent major changes during the evaluation process, or whether the development and/or content was “frozen” during the trial. Describe dynamic components such as news feeds or changing content which may have an impact on the replicability of the intervention (for unexpected events see item 3b).

	1	2	3	4	5	
subitem not at all important	○	○	○	◉	○	essential

Clear selection

Does your paper address subitem 5-iii?

Copy and paste relevant sections from the manuscript (include quotes in quotation marks "like this" to indicate direct quotes from your manuscript), or elaborate on this item by providing additional information not in the ms, or briefly explain why the item is not applicable/relevant for your study

No major changes to the app or issues with recruitment to date.

5-iv) Quality assurance methods

Provide information on quality assurance methods to ensure accuracy and quality of information provided [1], if applicable.

	1	2	3	4	5	
subitem not at all important	○	○	○	○	◉	essential

Clear selection

Does your paper address subitem 5-iv?

Copy and paste relevant sections from the manuscript (include quotes in quotation marks "like this" to indicate direct quotes from your manuscript), or elaborate on this item by providing additional information not in the ms, or briefly explain why the item is not applicable/relevant for your study

Data collection is not complete for the current manuscript.

5-v) Ensure replicability by publishing the source code, and/or providing screenshots/screen-capture video, and/or providing flowcharts of the algorithms used

Ensure replicability by publishing the source code, and/or providing screenshots/screen-capture video, and/or providing flowcharts of the algorithms used. Replicability (i.e., other researchers should in principle be able to replicate the study) is a hallmark of scientific reporting.

	1	2	3	4	5	
subitem not at all important	○	○	○	○	●	essential

Clear selection

Does your paper address subitem 5-v?

Copy and paste relevant sections from the manuscript (include quotes in quotation marks "like this" to indicate direct quotes from your manuscript), or elaborate on this item by providing additional information not in the ms, or briefly explain why the item is not applicable/relevant for your study

Data collection and analyses are not complete as the current manuscript only provides a protocol for a forthcoming RCT.

5-vi) Digital preservation

Digital preservation: Provide the URL of the application, but as the intervention is likely to change or disappear over the course of the years; also make sure the intervention is archived (Internet Archive, webcitation.org, and/or publishing the source code or screenshots/videos alongside the article). As pages behind login screens cannot be archived, consider creating demo pages which are accessible without login.

	1	2	3	4	5	
subitem not at all important	○	○	○	○	◉	essential

Clear selection

Does your paper address subitem 5-vi?

Copy and paste relevant sections from the manuscript (include quotes in quotation marks "like this" to indicate direct quotes from your manuscript), or elaborate on this item by providing additional information not in the ms, or briefly explain why the item is not applicable/relevant for your study

"The UseIt! system includes a cross-platform mHealth app that runs on both iOS and Android devices."

5-vii) Access

Access: Describe how participants accessed the application, in what setting/context, if they had to pay (or were paid) or not, whether they had to be a member of specific group. If known, describe how participants obtained “access to the platform and Internet” [1]. To ensure access for editors/reviewers/readers, consider to provide a “backdoor” login account or demo mode for reviewers/readers to explore the application (also important for archiving purposes, see vi).

	1	2	3	4	5	
subitem not at all important	○	○	○	○	◉	essential

Clear selection

Does your paper address subitem 5-vii? *

Copy and paste relevant sections from the manuscript (include quotes in quotation marks "like this" to indicate direct quotes from your manuscript), or elaborate on this item by providing additional information not in the ms, or briefly explain why the item is not applicable/relevant for your study

"The UseIt! system includes a free cross-platform mHealth app that runs on both iOS and Android devices. The app is securely connected to a portal where app feature usage is stored. The portal was designed for the research team to track and monitor usage of the app by parents. The app contains six features: (1) a troubleshooting guide that provides detailed skill recommendations for problem situations, (2) a behavior diary for tracking behaviors and skills used each day, (3) a digital library that provides definitions and instructions for each skill, (4) a point counter for parents to award points to their children, (5) a skills-alarm for reminding parents to practice the various skills, and (6) a timer for use with parenting skills (e.g., time-outs, managing screen time, routines). Users can examine diary entries, view points awarded, and set the skills-alarm through the app."

5-viii) Mode of delivery, features/functionalities/components of the intervention and comparator, and the theoretical framework

Describe mode of delivery, features/functionalities/components of the intervention and comparator, and the theoretical framework [6] used to design them (instructional strategy [1], behaviour change techniques, persuasive features, etc., see e.g., [7, 8] for terminology). This includes an in-depth description of the content (including where it is coming from and who developed it) [1],” whether [and how] it is tailored to individual circumstances and allows users to track their progress and receive feedback” [6]. This also includes a description of communication delivery channels and – if computer-mediated communication is a component – whether communication was synchronous or asynchronous [6]. It also includes information on presentation strategies [1], including page design principles, average amount of text on pages, presence of hyperlinks to other resources, etc. [1].

	1	2	3	4	5	
subitem not at all important	○	○	○	○	◉	essential

Clear selection

Does your paper address subitem 5-viii? *

Copy and paste relevant sections from the manuscript (include quotes in quotation marks "like this" to indicate direct quotes from your manuscript), or elaborate on this item by providing additional information not in the ms, or briefly explain why the item is not applicable/relevant for your study

"UseIt! Smartphone Application
The UseIt! system includes a free cross-platform mHealth app that runs on both iOS and Android devices. The app is securely connected to a portal where app feature usage is stored. The portal was designed for the research team to track and monitor usage of the app by parents. The app contains six features: (1) a troubleshooting guide that provides detailed skill recommendations for problem situations, (2) a behavior diary for tracking behaviors and skills used each day, (3) a digital library that provides definitions and instructions for each skill, (4) a point counter for parents to award points to their children, (5) a skills-alarm for reminding parents to practice the various skills, and (6) a timer for use with parenting skills (e.g., time-outs, managing screen time, routines). Users can examine diary entries, view points awarded, and set the skills-alarm through the app. See Figure 1 for

diary entries, view points awarded, and set the skills alarm through the app. See Figure 1 for a screenshot of the App home page. [Insert Figure 1 here]
Troubleshooting Guide
The UseIt! troubleshooting guide contains information to help parents effectively respond to problem-behaviors. Parents are presented with a list of potential negative behaviors (e.g. bullied or fought). After selecting a behavior, appropriate skill options (e.g. time-out) are displayed with tips to effectively apply each skill. Once a skill has been used and the behavior has stopped, parents are reminded to praise their child for positive behaviors. See Figure 2. [Insert Figure 2 here]
Behavior Diary
The behavior diary cues participants (via a notification) to complete a series of questions about behaviors and PMT/CBT skills used each day. The results are displayed graphically and can be reviewed by the user to track progress over time. This allows the user to keep track of what skills the family has tried for different behaviors (both positive and negative child behaviors) and which ones have been helpful in various contexts. See Figure 3. [Insert Figure 3 here]
Digital Library
The UseIt! digital library provides detailed information about using strategies for positive and negative child behavior. Each skill is defined and presented with tips for how and when to effectively use each skill. The digital library contains more information than the troubleshooting guide and is designed as an information source for reviewing PMT/CBT topics. See Figure 4. [Insert Figure 4 here]
Point Counter
Treatment for disruptive behavior disorders typically includes prizes for treatment adherence, positive behaviors, and skill use. Parents can award points to children for target behaviors (e.g. cleaning dishes) and skill utilization. The UseIt! point counter features an on-screen button (“Give your child a point”) which parents press to reward their child with a point. The feature functions as a digital rewards program that parents can use to keep track of points and reward their children. See Figure 1 for a view of the point counter window.
Skills Alarm
Skills alarms can be set at any time via the app. Users can set dates and times for notifications to activate. These notifications remind parents and children to practice specific skills throughout the week (e.g. “remember to praise your child”). Parents are able to view a list of active and inactive alarms. See Figure 5. [Insert Figure 5 here]
Timer
Timers can be set for use with a variety of skills (e.g., time-outs, screen time management, routines) to promote use of the skills."

5-ix) Describe use parameters

Describe use parameters (e.g., intended “doses” and optimal timing for use). Clarify what instructions or recommendations were given to the user, e.g., regarding timing, frequency, heaviness of use, if any, or was the intervention used ad libitum.

	1	2	3	4	5	
subitem not at all important	○	○	○	○	◉	essential

Clear selection

Does your paper address subitem 5-ix?

Copy and paste relevant sections from the manuscript (include quotes in quotation marks "like this" to indicate direct quotes from your manuscript), or elaborate on this item by providing additional information not in the ms, or briefly explain why the item is not applicable/relevant for your study

"Standalone UseIt! App Condition

Participants are assigned to the UseIt! App as a standalone intervention for four months.

UseIt! App + Coaching Condition

Participants are assigned to the UseIt! App and are provided with an mHealth “Coach” for four months. The primary objective of the coaching condition is to promote engagement with the UseIt! mHealth platform. The mHealth coach will be a bachelor’s-level paraprofessional with a degree in psychology or an allied discipline (e.g., social work) who will provide support to parents in the coach-assisted condition. We selected a bachelor’s-level coach over a Master’s-level coach to enhance the scalability of this intervention condition. The coach will use the Supportive Accountability (SA) coaching model using the training guidelines outlined by Dobke and colleagues [20]. During the intervention phase of the study, parents will be contacted by the coach once per week by phone and also allowed to contact the coach during regular business hours. The coach will provide motivation and accountability but will not provide therapeutic/clinical support. The primary goal of the coach is to increase participant engagement with the UseIt! mHealth system. Specific coaching content/tasks will include, 1) social support, 2) promoting engagement with the app, 3) goal setting 4) monitoring progress, and 5) encouragement/motivation. Parents will be provided with appropriate referrals for any crises. The coach will be instructed to respond to questions that lie outside the domains of motivation and accountability (i.e., content-

specific therapeutic support) by redirecting the parent to the content-specific app features (i.e., Troubleshooting and Library). Only the parent(s) will interact with the coach. To maintain the scalability of the condition, the target time spent with each family will be 15-30 minutes per week. The coach will maintain a “coach-log” to track the frequency, duration, and content of contact with each participant.
Control Condition (Smiling Mind app)
Parents in the control app condition will be assigned to use a mindfulness app called Smiling Mind [21] for four months. We selected a mindfulness app because it is an active control condition, but one that we do not expect to engage the same treatment targets as the UseIt! mHealth system. Meta-analytic findings indicate that mindfulness-based interventions for school-age children are associated with medium to large effect sizes for disruptive behaviors [22]. For parents, dispositional mindfulness has been found to be associated with lower rates of children’s externalizing and internalizing problems [23]; and a mindfulness-based program was associated with decreased parent reports of child ADHD symptoms and decreased parental stress [24]. Other studies with youth found that a combined approach (parent and youth mindfulness training) improved externalizing problems and attention [25].As an active control condition, we expect that a mindfulness app will likely have some influence on parenting and child behavior. This will provide us with a rigorous control condition while also allowing us to test the specificity of target engagement. We expect the Smiling Mind app will only enhance mindfulness, but not PMT/CBT skills (other than mindfulness). We selected the Smiling Mind app in particular because: 1) it can be downloaded at no cost, 2) it is available for both Android and Apple (iPhone) devices, and 3) app-use can be tracked."

5-x) Clarify the level of human involvement

Clarify the level of human involvement (care providers or health professionals, also technical assistance) in the e-intervention or as co-intervention (detail number and expertise of professionals involved, if any, as well as “type of assistance offered, the timing and frequency of the support, how it is initiated, and the medium by which the assistance is delivered”. It may be necessary to distinguish between the level of human involvement required for the trial, and the level of human involvement required for a routine application outside of a RCT setting (discuss under item 21 – generalizability).

	1	2	3	4	5	
subitem not at all important	○	○	○	○	◉	essential

Clear selection

Does your paper address subitem 5-x?

Copy and paste relevant sections from the manuscript (include quotes in quotation marks "like this" to indicate direct quotes from your manuscript), or elaborate on this item by providing additional information not in the ms, or briefly explain why the item is not applicable/relevant for your study

"UseIt! App + Coaching Condition
Participants are assigned to the UseIt! App and are provided with an mHealth “Coach” for four months. The primary objective of the coaching condition is to promote engagement with the UseIt! mHealth platform. The mHealth coach will be a bachelor’s-level paraprofessional with a degree in psychology or an allied discipline (e.g., social work) who will provide support to parents in the coach-assisted condition. We selected a bachelor’s-level coach over a Master’s-level coach to enhance the scalability of this intervention condition. The coach will use the Supportive Accountability (SA) coaching model using the training guidelines outlined by Dobke and colleagues [20]. During the intervention phase of the study, parents will be contacted by the coach once per week by phone and also allowed to contact the coach during regular business hours. The coach will provide motivation and accountability but will not provide therapeutic/clinical support. The primary goal of the coach is to increase participant engagement with the UseIt! mHealth system. Specific coaching content/tasks will include, 1) social support, 2) promoting engagement with the app, 3) goal setting 4) monitoring progress, and 5) encouragement/motivation. Parents will be provided with appropriate referrals for any crises. The coach will be instructed to respond to questions that lie outside the domains of motivation and accountability (i.e., content-specific therapeutic support) by redirecting the parent to the content-specific app features (i.e., Troubleshooting and Library). Only the parent(s) will interact with the coach. To maintain the scalability of the condition, the target time spent with each family will be 15-30 minutes per week. The coach will maintain a “coach-log” to track the frequency, duration, and content of contact with each participant."

5-xi) Report any prompts/reminders used

Report any prompts/reminders used: Clarify if there were prompts (letters, emails, phone calls, SMS) to use the application, what triggered them, frequency etc. It may be necessary to distinguish between the level of prompts/reminders required for the trial, and the level of prompts/reminders for a routine application outside of a RCT setting (discuss under item 21 – generalizability).

	1	2	3	4	5	
subitem not at all important	○	○	○	○	●	essential

Clear selection

Does your paper address subitem 5-xi? *

Copy and paste relevant sections from the manuscript (include quotes in quotation marks "like this" to indicate direct quotes from your manuscript), or elaborate on this item by providing additional information not in the ms, or briefly explain why the item is not applicable/relevant for your study

Parents in the UseIt! app conditions were sent daily reminders to use the app. " The behavior diary cues participants (via a notification) to complete a series of questions about behaviors and PMT/CBT skills used each day."

5-xii) Describe any co-interventions (incl. training/support)

Describe any co-interventions (incl. training/support): Clearly state any interventions that are provided in addition to the targeted eHealth intervention, as ehealth intervention may not be designed as stand-alone intervention. This includes training sessions and support [1]. It may be necessary to distinguish between the level of training required for the trial, and the level of training for a routine application outside of a RCT setting (discuss under item 21 – generalizability.

	1	2	3	4	5	
subitem not at all important	○	○	○	○	◉	essential

Clear selection

Does your paper address subitem 5-xii? *

Copy and paste relevant sections from the manuscript (include quotes in quotation marks "like this" to indicate direct quotes from your manuscript), or elaborate on this item by providing additional information not in the ms, or briefly explain why the item is not applicable/relevant for your study

"Parents assigned to the Coach condition will be walked through the initial setup and login process, along with a brief training on how to use the applications over the phone. Families can be set up and trained in approximately 30 minutes. If parents cannot be reached after three weeks, instructions are sent via email. Parents assigned to the standalone UseIt! app condition are sent a tutorial video with the same information."

6a) Completely defined pre-specified primary and secondary outcome measures, including how and when they were assessed

Does your paper address CONSORT subitem 6a? *

Copy and paste relevant sections from the manuscript (include quotes in quotation marks "like this" to indicate direct quotes from your manuscript), or elaborate on this item by providing additional information not in the ms, or briefly explain why the item is not applicable/relevant for your study

"Primary Outcome
PMT/CBT Skill Knowledge
The Knowledge of Effective Parenting Test (KEPT[26]) is a 21-item measure of parental knowledge of effective parenting skills. The measure was developed as a potential treatment target for evidence-based psychosocial treatments of disruptive behaviors in children. The KEPT assesses parental knowledge of domains including praise, rewards/point systems, attending and ignoring, commands/expectations, consequences, and time-outs. Parents are presented with a series of video and text-based parenting scenarios and questions with four multiple-choice response options. Scores range from 0 to 21. The measure has good reliability (Cronbach's alpha = .84). The measure has also demonstrated convergent validity with other measures of parenting knowledge and parenting related constructs (e.g., child behavior, parental psychopathology).
Secondary Outcomes
Symptom Severity
Vanderbilt Assessment Scale-Parent Report (VASPR [27]). The VASPR is a 55-item parent-report screen for Attention-Deficit/Hyperactivity Disorder (ADHD), Oppositional Defiant Disorder (ODD), and Conduct Disorder (CD). It also includes seven items on internalizing symptoms and eight items on school performance and social functioning. Symptom items are rated using a 4-point scale and the performance items are rated on a 5-point scale. The measure has Cronbach's alphas ranging from .79 to .95 and strong evidence of construct validity.
Parenting Practices
The Alabama Parenting Questionnaire (APQ [28]) is a 42-item measure that assesses five dimensions of parenting: (1) positive involvement, (2) monitoring, (3) positive discipline, (4) consistency, and (5) corporal punishment, using a 5-point scale ranging from 1 to 5. The internal consistency of the scale is acceptable with alphas for the five domains ranging up to .80. The measure has well-established construct validity.
Parent Depression
Patient Health Questionnaire-8 (PHQ-8 [29]). The PHQ-8 measures symptoms of depression using a 4-point scale from "not at all" to "nearly every day." Total scores range from 0 to 24. The measure has a reported Cronbach's alpha of .82 and strong construct validity.
Parent Anxiety
General Anxiety Disorder-7 (GAD-7 [30]). The GAD-7 is a 7-item measure of anxiety. Items are rated on a 4-point scale from "not at all" to "nearly every day." The measure includes an item to assess the duration of anxiety symptoms. The measure has excellent internal

item to assess the duration of anxiety symptoms. The measure has excellent internal consistency (Cronbach's alpha = 0.92), good test-retest reliability (intraclass correlation = 0.83), and strong convergent validity with other measures of anxiety.

Parenting Stress

The Parental Stress Scale (PSS [31]) is an 18-item measure of stress related to parental experiences. Items are rated on a 5-point scale from "strongly disagree" to "strongly agree". Scores range from 18 to 90. The internal consistency of the scale is acceptable with a Cronbach's alpha of 0.83, a test-retest reliability of 0.81 (intraclass correlation, ICC), and strong convergent validity of both other parental stress measures (i.e., Parental Stress Index), and other measures related to parenting stress (e.g., loneliness, marital satisfaction, social support).

Social Support

The Social Provisions Scale (SPS [32]) is a 24-item measure that assesses six dimensions of support, including attachment, social integration, opportunity for nurturance, reassurance of worth, reliable alliance, and guidance. The measure uses a five-point scale ranging from 1 to 4, strongly disagree to strongly agree. The measure has been validated across samples, with Cronbach's alphas ranging from .65 to .76 for the four subscales, and a total reliability estimate of .91. The measure also demonstrated convergent validity with related measures of social support.

PMT/CBT Skill Use

The Parenting Skill Use Diary (PSUD [33]) assesses daily use of parenting skills in everyday parenting contexts (e.g., child sharing and helping, hitting, fighting). Respondents are presented with a checklist of behaviors to report on for the past week. For each behavior they select, they are next asked to identify which skills (e.g., praise, reward, time-out, loss-of-privilege) they used in responding to the behaviors. The instrument has demonstrated the ability to capture significant between person variability in appropriate PMT skills. A weekly summary score discriminated between parents/guardians whose children screened positive versus negative for Conduct Disorder (AUC = .72) and Oppositional Defiant Disorder (AUC = .70).

App Use

Parents assigned to both UseIt! conditions (i.e., standalone app and coach conditions) will have their data stored on the secure portal, accessible to the research team. We collect the behavior diary tracked by parents, which displays data on child behavior (both positive and negative) along with CBT/PMT skills. We are also collecting data on time spent on the app along with modules accessed. App usage (e.g., modules used, time spent) is also tracked for the Smiling Mind app (control condition).

Mindfulness

The Mindful Attention Awareness Scale (MAAS [34]) is a 15-item scale designed to assess core characteristics of mindfulness. Parents are asked how often they are engaging in a variety of mindfulness-related behaviors, such as finding it difficult to stay focused on what's happening in the present moment, finding themselves preoccupied with the future or past, and not noticing feelings of physical tension or discomfort until the really grab their attention. The measure's response scale ranges from 1 (almost always) to 6 (almost never). The measure is scored as an average of all 15 items. The measure has adequate validity

The measure is scored as an average of all 15 items. The measure has adequate validity (Cronbach’s alpha = .87 and convergent validity with other measures of mindfulness (e.g., mood disturbances, stress).
Service Use
Service Assessment for Children and Adolescents (SACA [35]) Abbreviated. An abbreviated version of the SACA (25-items) was used to measure mental health service utilization. The SACA asks about various inpatient and outpatient treatment services for mental/behavioral health problems that have been utilized by the child in the past six months [35]. Most items are yes/no questions. The measure is a widely used research tool with strong evidence of reliability and validity.
Supportive Accountability
The Supportive Accountability Index (SAI [36]) is an 8-item measure of how well a given platform functioned to help keep parents accountable to accomplish a given goal. The measure was included to assess the effectiveness of the Coach in the coach condition of the app in helping to keep parents accountable with skill learning and use. Items are rated on a 1-7 scale, from strongly disagree to strongly agree. Total scores are summed, ranging from 8-56. The measure has acceptable validity (Cronbach’s alpha = .68) and good convergent and divergent validity.
Usability
Post-Study System Usability Questionnaire (PSSUQ [37]). The 19-item PSSUQ will be used to assess overall user satisfaction with the UseIt! apps. Internal consistency of the PSSUQ is excellent (α = 0.91 to 0.96).
Technological Literacy
The Technological Self-Assessment Scale (TSAT [38]) is a 13-item parent report screen for technological ability. The measure was created for the current study by the research team to provide an indicator of parent knowledge and experience with their computers and phones. For example, items ask parents if they know how to search for information on the internet, if they have ever downloaded an app, and if they have social media accounts. Items are scored Yes/No, and scores range from 0-13. [Insert Table 1 here]"

6a-i) Online questionnaires: describe if they were validated for online use and apply CHERRIES items to describe how the questionnaires were designed/deployed

If outcomes were obtained through online questionnaires, describe if they were validated for online use and apply CHERRIES items to describe how the questionnaires were designed/deployed [9].

	1	2	3	4	5	
subitem not at all important	○	○	○	○	◉	essential

Clear selection

Does your paper address subitem 6a-i?

Copy and paste relevant sections from manuscript text

We used measures commonly used in the literature. All measures were validated, which is described in the method, below.
"Primary Outcome
PMT/CBT Skill Knowledge
The Knowledge of Effective Parenting Test (KEPT[26]) is a 21-item measure of parental knowledge of effective parenting skills. The measure was developed as a potential treatment target for evidence-based psychosocial treatments of disruptive behaviors in children. The KEPT assesses parental knowledge of domains including praise, rewards/point systems, attending and ignoring, commands/expectations, consequences, and time-outs. Parents are presented with a series of video and text-based parenting scenarios and questions with four multiple-choice response options. Scores range from 0 to 21. The measure has good reliability (Cronbach's alpha = .84). The measure has also demonstrated convergent validity with other measures of parenting knowledge and parenting related constructs (e.g., child behavior, parental psychopathology).
Secondary Outcomes
Symptom Severity
Vanderbilt Assessment Scale-Parent Report (VASPR [27]). The VASPR is a 55-item parent-report screen for Attention-Deficit/Hyperactivity Disorder (ADHD), Oppositional Defiant Disorder (ODD), and Conduct Disorder (CD). It also includes seven items on internalizing symptoms and eight items on school performance and social functioning. Symptom items are rated using a 4-point scale and the performance items are rated on a 5-point scale. The measure has Cronbach's alphas ranging from .79 to .95 and strong evidence of construct

measure has Cronbach's alphas ranging from .79 to .9[illegible] and strong evidence of construct validity.

Parenting Practices

The Alabama Parenting Questionnaire (APQ [28]) is a 42-item measure that assesses five dimensions of parenting: (1) positive involvement, (2) monitoring, (3) positive discipline, (4) consistency, and (5) corporal punishment, using a 5-point scale ranging from 1 to 5. The internal consistency of the scale is acceptable with alphas for the five domains ranging up to .80. The measure has well-established construct validity.

Parent Depression

Patient Health Questionnaire-8 (PHQ-8 [29]). The PHQ-8 measures symptoms of depression using a 4-point scale from “not at all” to “nearly every day.” Total scores range from 0 to 24. The measure has a reported Cronbach’s alpha of .82 and strong construct validity.

Parent Anxiety

General Anxiety Disorder-7 (GAD-7 [30]). The GAD-7 is a 7-item measure of anxiety. Items are rated on a 4-point scale from “not at all” to “nearly every day.” The measure includes an item to assess the duration of anxiety symptoms. The measure has excellent internal consistency (Cronbach’s alpha = 0.92), good test-retest reliability (intraclass correlation = 0.83), and strong convergent validity with other measures of anxiety.

Parenting Stress

The Parental Stress Scale (PSS [31]) is an 18-item measure of stress related to parental experiences. Items are rated on a 5-point scale from “strongly disagree” to “strongly agree”. Scores range from 18 to 90. The internal consistency of the scale is acceptable with a Cronbach’s alpha of 0.83, a test-retest reliability of 0.81 (intraclass correlation, ICC), and strong convergent validity of both other parental stress measures (i.e., Parental Stress Index), and other measures related to parenting stress (e.g., loneliness, marital satisfaction, social support).

Social Support

The Social Provisions Scale (SPS [32]) is a 24-item measure that assesses six dimensions of support, including attachment, social integration, opportunity for nurturance, reassurance of worth, reliable alliance, and guidance. The measure uses a five-point scale ranging from 1 to 4, strongly disagree to strongly agree. The measure has been validated across samples, with Cronbach’s alphas ranging from .65 to .76 for the four subscales, and a total reliability estimate of .91. The measure also demonstrated convergent validity with related measures of social support.

PMT/CBT Skill Use

The Parenting Skill Use Diary (PSUD [33]) assesses daily use of parenting skills in everyday parenting contexts (e.g., child sharing and helping, hitting, fighting). Respondents are presented with a checklist of behaviors to report on for the past week. For each behavior they select, they are next asked to identify which skills (e.g., praise, reward, time-out, loss-of-privilege) they used in responding to the behaviors. The instrument has demonstrated the ability to capture significant between person variability in appropriate PMT skills. A weekly summary score discriminated between parents/guardians whose children screened positive versus negative for Conduct Disorder (AUC = .72) and Oppositional Defiant Disorder (AUC = [illegible])

.70).
App Use
Parents assigned to both UseIt! conditions (i.e., standalone app and coach conditions) will have their data stored on the secure portal, accessible to the research team. We collect the behavior diary tracked by parents, which displays data on child behavior (both positive and negative) along with CBT/PMT skills. We are also collecting data on time spent on the app along with modules accessed. App usage (e.g., modules used, time spent) is also tracked for the Smiling Mind app (control condition).
Mindfulness
The Mindful Attention Awareness Scale (MAAS [34]) is a 15-item scale designed to assess core characteristics of mindfulness. Parents are asked how often they are engaging in a variety of mindfulness-related behaviors, such as finding it difficult to stay focused on what's happening in the present moment, finding themselves preoccupied with the future or past, and not noticing feelings of physical tension or discomfort until the really grab their attention. The measure's response scale ranges from 1 (almost always) to 6 (almost never). The measure is scored as an average of all 15 items. The measure has adequate validity (Cronbach's alpha = .87 and convergent validity with other measures of mindfulness (e.g., mood disturbances, stress).
Service Use
Service Assessment for Children and Adolescents (SACA [35]) Abbreviated. An abbreviated version of the SACA (25-items) was used to measure mental health service utilization. The SACA asks about various inpatient and outpatient treatment services for mental/behavioral health problems that have been utilized by the child in the past six months [35]. Most items are yes/no questions. The measure is a widely used research tool with strong evidence of reliability and validity.
Supportive Accountability
The Supportive Accountability Index (SAI [36]) is an 8-item measure of how well a given platform functioned to help keep parents accountable to accomplish a given goal. The measure was included to assess the effectiveness of the Coach in the coach condition of the app in helping to keep parents accountable with skill learning and use. Items are rated on a 1-7 scale, from strongly disagree to strongly agree. Total scores are summed, ranging from 8-56. The measure has acceptable validity (Cronbach's alpha = .68) and good convergent and divergent validity.
Usability
Post-Study System Usability Questionnaire (PSSUQ [37]). The 19-item PSSUQ will be used to assess overall user satisfaction with the UseIt! apps. Internal consistency of the PSSUQ is excellent (α = 0.91 to 0.96).
Technological Literacy
The Technological Self-Assessment Scale (TSAT [38]) is a 13-item parent report screen for technological ability. The measure was created for the current study by the research team to provide an indicator of parent knowledge and experience with their computers and phones. For example, items ask parents if they know how to search for information on the internet, if they have ever downloaded an app, and if they have social media accounts. Items are
[illegible]

scored Yes/No, and scores range from 0-13. [Insert Table 1 here]"

6a-ii) Describe whether and how "use" (including intensity of use/dosage) was defined/measured/monitored

Describe whether and how "use" (including intensity of use/dosage) was defined/measured/monitored (logins, logfile analysis, etc.). Use/adoption metrics are important process outcomes that should be reported in any ehealth trial.

	1	2	3	4	5	
subitem not at all important	○	○	○	○	◉	essential

Clear selection

Does your paper address subitem 6a-ii?

Copy and paste relevant sections from manuscript text

"App Use
Parents assigned to both UseIt! conditions (i.e., standalone app and coach conditions) will have their data stored on the secure portal, accessible to the research team. We collect the behavior diary tracked by parents, which displays data on child behavior (both positive and negative) along with CBT/PMT skills. We are also collecting data on time spent on the app along with modules accessed. App usage (e.g., modules used, time spent) is also tracked for the Smiling Mind app (control condition)."

6a-iii) Describe whether, how, and when qualitative feedback from participants was obtained

Describe whether, how, and when qualitative feedback from participants was obtained (e.g., through emails, feedback forms, interviews, focus groups).

	1	2	3	4	5	
subitem not at all important	○	○	○	○	◉	essential

Clear selection

Does your paper address subitem 6a-iii?

Copy and paste relevant sections from manuscript text

Trial is not complete as this is a protocol, not a complete RCT. Thus, we do not have qualitative information.

6b) Any changes to trial outcomes after the trial commenced, with reasons

Does your paper address CONSORT subitem 6b? *

Copy and paste relevant sections from the manuscript (include quotes in quotation marks "like this" to indicate direct quotes from your manuscript), or elaborate on this item by providing additional information not in the ms, or briefly explain why the item is not applicable/relevant for your study

NA, as the current manuscript describes a protocol rather than a complete RCT.

7a) How sample size was determined

NPT: When applicable, details of whether and how the clustering by care provides or centers was addressed

7a-i) Describe whether and how expected attrition was taken into account when calculating the sample size

Describe whether and how expected attrition was taken into account when calculating the sample size.

	1	2	3	4	5	
subitem not at all important	○	○	○	○	◉	essential

Clear selection

Does your paper address subitem 7a-i?

Copy and paste relevant sections from manuscript title (include quotes in quotation marks "like this" to indicate direct quotes from your manuscript), or elaborate on this item by providing additional information not in the ms, or briefly explain why the item is not applicable/relevant for your study

"Sample Size Determination
Power analyses were conducted in PASS version 13.0.8 to ensure that the sample size is adequate to test the primary hypotheses with adequate statistical power. All analyses assumed 0.80 power and two-sided tests. Estimated sample sizes are determined based on an assumed 5% attrition/missing data at post-treatment (102 per group) and 10% total attrition at 6-months (97 per group). Using an ANCOVA approach to test for differences across three groups at post-treatment (n = 108 each) and assuming R2 = 0.20 from five covariates, we expect 0.80 power (α = 0.05) to detect an effect size difference of Cohen's f = 0.16 among the 3 groups, f = 0.14 (d = 0.28) between both UseIt! treatment groups versus the control group, and f = 0.18 (d = 0.36) between any two groups."

7b) When applicable, explanation of any interim analyses and stopping guidelines

Does your paper address CONSORT subitem 7b? *

Copy and paste relevant sections from the manuscript (include quotes in quotation marks "like this" to indicate direct quotes from your manuscript), or elaborate on this item by providing additional information not in the ms, or briefly explain why the item is not applicable/relevant for your study

NA as the current trial has not completed data collection, and we have not completed any analyses.

8a) Method used to generate the random allocation sequence

NPT: When applicable, how care providers were allocated to each trial group

Does your paper address CONSORT subitem 8a? *

Copy and paste relevant sections from the manuscript (include quotes in quotation marks "like this" to indicate direct quotes from your manuscript), or elaborate on this item by providing additional information not in the ms, or briefly explain why the item is not applicable/relevant for your study

"We use stratified randomization to ensure that the groups are equivalent on key clinical features (screening severity and referral source (i.e., Pitt + Me or BuildClinical)."

8b) Type of randomisation; details of any restriction (such as blocking and block size)

Does your paper address CONSORT subitem 8b? *

Copy and paste relevant sections from the manuscript (include quotes in quotation marks "like this" to indicate direct quotes from your manuscript), or elaborate on this item by providing additional information not in the ms, or briefly explain why the item is not applicable/relevant for your study

"We use stratified randomization to ensure that the groups are equivalent on key clinical features (screening severity and referral source (i.e., Pitt + Me or BuildClinical)."

9) Mechanism used to implement the random allocation sequence (such as sequentially numbered containers), describing any steps taken to conceal the sequence until interventions were assigned

Does your paper address CONSORT subitem 9? *

Copy and paste relevant sections from the manuscript (include quotes in quotation marks "like this" to indicate direct quotes from your manuscript), or elaborate on this item by providing additional information not in the ms, or briefly explain why the item is not applicable/relevant for your study

"We use stratified randomization to ensure that the groups are equivalent on key clinical features (screening severity and referral source (i.e., Pitt + Me or BuildClinical)."

10) Who generated the random allocation sequence, who enrolled participants, and who assigned participants to interventions

Does your paper address CONSORT subitem 10? *

Copy and paste relevant sections from the manuscript (include quotes in quotation marks "like this" to indicate direct quotes from your manuscript), or elaborate on this item by providing additional information not in the ms, or briefly explain why the item is not applicable/relevant for your study

"We use stratified randomization to ensure that the groups are equivalent on key clinical features (screening severity and referral source (i.e., Pitt + Me or BuildClinical)."

11a) If done, who was blinded after assignment to interventions (for example, participants, care providers, those assessing outcomes) and how

NPT: Whether or not administering co-interventions were blinded to group assignment

11a-i) Specify who was blinded, and who wasn’t

Specify who was blinded, and who wasn’t. Usually, in web-based trials it is not possible to blind the participants [1, 3] (this should be clearly acknowledged), but it may be possible to blind outcome assessors, those doing data analysis or those administering co-interventions (if any).

	1	2	3	4	5	
subitem not at all important	○	○	○	◉	○	essential

Clear selection

Does your paper address subitem 11a-i? *

Copy and paste relevant sections from the manuscript (include quotes in quotation marks "like this" to indicate direct quotes from your manuscript), or elaborate on this item by providing additional information not in the ms, or briefly explain why the item is not applicable/relevant for your study

As the trial is mobile-based, participants are not blinded to their condition assignment.

11a-ii) Discuss e.g., whether participants knew which intervention was the “intervention of interest” and which one was the “comparator”

Informed consent procedures (4a-ii) can create biases and certain expectations - discuss e.g., whether participants knew which intervention was the “intervention of interest” and which one was the “comparator”.

	1	2	3	4	5	
subitem not at all important	○	○	○	○	○	essential

Does your paper address subitem 11a-ii?

Copy and paste relevant sections from the manuscript (include quotes in quotation marks "like this" to indicate direct quotes from your manuscript), or elaborate on this item by providing additional information not in the ms, or briefly explain why the item is not applicable/relevant for your study

Parents are not cued as to which intervention is the intervention of interest, clarified in the informed consent.

11b) If relevant, description of the similarity of interventions
(this item is usually not relevant for ehealth trials as it refers to similarity of a placebo or sham intervention to a active medication/intervention)

Does your paper address CONSORT subitem 11b? *

Copy and paste relevant sections from the manuscript (include quotes in quotation marks "like this" to indicate direct quotes from your manuscript), or elaborate on this item by providing additional information not in the ms, or briefly explain why the item is not applicable/relevant for your study

"Control Condition (Smiling Mind app)
Parents in the control app condition will be assigned to use a mindfulness app called Smiling Mind [21] for four months. We selected a mindfulness app because it is an active control condition, but one that we do not expect to engage the same treatment targets as the UseIt! mHealth system. Meta-analytic findings indicate that mindfulness-based interventions for school-age children are associated with medium to large effect sizes for disruptive behaviors [22]. For parents, dispositional mindfulness has been found to be associated with lower rates of children’s externalizing and internalizing problems [23]; and a mindfulness-based program was associated with decreased parent reports of child ADHD symptoms and decreased parental stress [24]. Other studies with youth found that a combined approach (parent and youth mindfulness training) improved externalizing problems and attention [25].As an active control condition, we expect that a mindfulness app will likely have some influence on parenting and child behavior. This will provide us with a rigorous control condition while also allowing us to test the specificity of target engagement. We expect the Smiling Mind app will only enhance mindfulness, but not PMT/CBT skills (other than mindfulness). We selected the Smiling Mind app in particular because: 1) it can be downloaded at no cost, 2) it is available for both Android and Apple (iPhone) devices, and 3) app-use can be tracked.
UseIt! Smartphone Application
The UseIt! system includes a free cross-platform mHealth app that runs on both iOS and Android devices. The app is securely connected to a portal where app feature usage is stored. The portal was designed for the research team to track and monitor usage of the app by parents. The app contains six features: (1) a troubleshooting guide that provides detailed skill recommendations for problem situations, (2) a behavior diary for tracking behaviors and skills used each day, (3) a digital library that provides definitions and instructions for each skill, (4) a point counter for parents to award points to their children, (5) a skills-alarm for reminding parents to practice the various skills, and (6) a timer for use with parenting skills (e.g., time-outs, managing screen time, routines). Users can examine diary entries, view points awarded, and set the skills-alarm through the app. See Figure 1 for a screenshot of the App home page. [Insert Figure 1 here]"

12a) Statistical methods used to compare groups for primary and secondary outcomes
NPT: When applicable, details of whether and how the clustering by care providers or centers was addressed

Does your paper address CONSORT subitem 12a? *

Copy and paste relevant sections from the manuscript (include quotes in quotation marks "like this" to indicate direct quotes from your manuscript), or elaborate on this item by providing additional information not in the ms, or briefly explain why the item is not applicable/relevant for your study

"Primary Analyses
Our primary analytic strategy will use intent-to-treat (ITT) analyses. We will examine reasons for any missing data and perform multiple imputation (e.g., Multiple Imputation for Chained Equations, MICE [39]) for data missing at random. To evaluate the effectiveness of the UseIt! mHealth system as both a standalone (n = 108) and coach-assisted (n = 108) intervention compared to a control app condition (n = 108), we will regress the primary and secondary outcomes on the study condition. We will also perform a priori tests to compare both UseIt! groups to the control and to compare the coach-assisted and standalone UseIt! groups. Cohen's d effect sizes will be estimated for between-group differences as well as pre-post changes within each group.
Secondary Analyses
We will also test whether gains in knowledge of parenting skills are associated with symptom reduction. We will regress post-treatment knowledge of parenting skills on study condition, pre-treatment knowledge, and their interaction. We will also compare app usage across standalone and coach-assisted conditions and test whether the app usage indices are associated with target engagement and symptom reduction at post-treatment. App usage outcomes will include the number of each of the app features that are utilized, along with frequency and duration (in minutes). We will use generalized linear models with the appropriate link (e.g., log link for count data, identity link for continuous outcomes) to regress each usage outcome on study condition (standalone versus coach)."

12a-i) Imputation techniques to deal with attrition / missing values

Imputation techniques to deal with attrition / missing values: Not all participants will use the intervention/comparator as intended and attrition is typically high in ehealth trials. Specify how participants who did not use the application or dropped out from the trial were treated in the statistical analysis (a complete case analysis is strongly discouraged, and simple imputation techniques such as LOCF may also be problematic [4]).

	1	2	3	4	5	
subitem not at all important	○	○	○	○	◉	essential

Clear selection

Does your paper address subitem 12a-i? *

Copy and paste relevant sections from the manuscript (include quotes in quotation marks "like this" to indicate direct quotes from your manuscript), or elaborate on this item by providing additional information not in the ms, or briefly explain why the item is not applicable/relevant for your study

"Primary Analyses
Our primary analytic strategy will use intent-to-treat (ITT) analyses. We will examine reasons for any missing data and perform multiple imputation (e.g., Multiple Imputation for Chained Equations, MICE [39]) for data missing at random. To evaluate the effectiveness of the UseIt! mHealth system as both a standalone (n = 108) and coach-assisted (n = 108) intervention compared to a control app condition (n = 108), we will regress the primary and secondary outcomes on the study condition. We will also perform a priori tests to compare both UseIt! groups to the control and to compare the coach-assisted and standalone UseIt! groups. Cohen's d effect sizes will be estimated for between-group differences as well as pre-post changes within each group.
Secondary Analyses
We will also test whether gains in knowledge of parenting skills are associated with symptom reduction. We will regress post-treatment knowledge of parenting skills on study condition, pre-treatment knowledge, and their interaction. We will also compare app usage across standalone and coach-assisted conditions and test whether the app usage indices are associated with target engagement and symptom reduction at post-treatment. App usage outcomes will include the number of each of the app features that are utilized, along with frequency and duration (in minutes). We will use generalized linear models with the appropriate link (e.g., log link for count data, identity link for continuous outcomes) to regress each usage outcome on study condition (standalone versus coach)."

12b) Methods for additional analyses, such as subgroup analyses and adjusted analyses

Does your paper address CONSORT subitem 12b? *

Copy and paste relevant sections from the manuscript (include quotes in quotation marks "like this" to indicate direct quotes from your manuscript), or elaborate on this item by providing additional information not in the ms, or briefly explain why the item is not applicable/relevant for your study

"Primary Analyses
Our primary analytic strategy will use intent-to-treat (ITT) analyses. We will examine reasons for any missing data and perform multiple imputation (e.g., Multiple Imputation for Chained Equations, MICE [39]) for data missing at random. To evaluate the effectiveness of the UseIt! mHealth system as both a standalone (n = 108) and coach-assisted (n = 108) intervention compared to a control app condition (n = 108), we will regress the primary and secondary outcomes on the study condition. We will also perform a priori tests to compare both UseIt! groups to the control and to compare the coach-assisted and standalone UseIt! groups. Cohen’s d effect sizes will be estimated for between-group differences as well as pre-post changes within each group.
Secondary Analyses
We will also test whether gains in knowledge of parenting skills are associated with symptom reduction. We will regress post-treatment knowledge of parenting skills on study condition, pre-treatment knowledge, and their interaction. We will also compare app usage across standalone and coach-assisted conditions and test whether the app usage indices are associated with target engagement and symptom reduction at post-treatment. App usage outcomes will include the number of each of the app features that are utilized, along with frequency and duration (in minutes). We will use generalized linear models with the appropriate link (e.g., log link for count data, identity link for continuous outcomes) to regress each usage outcome on study condition (standalone versus coach)."

X26) REB/IRB Approval and Ethical Considerations [recommended as subheading under "Methods"] (not a CONSORT item)

X26-i) Comment on ethics committee approval

	1	2	3	4	5	
subitem not at all important	○	○	○	○	◉	essential

Clear selection

Does your paper address subitem X26-i?

Copy and paste relevant sections from the manuscript (include quotes in quotation marks "like this" to indicate direct quotes from your manuscript), or elaborate on this item by providing additional information not in the ms, or briefly explain why the item is not applicable/relevant for your study

"Ethical Considerations
Ethical approval has been obtained from the Institutional Review Board at the University of Pittsburgh (protocol # STUDY22030138Informed consent from parents is obtained by trained research So as not to artificially inflate the rates of app utilization, participants are not compensated for using the app but only for assessments completed. Participants in each condition are compensated $20 for baseline assessment completion, $40 for the post-assessment, and $60 for the six-month follow-up assessment. All study data are deidentified and stored securely. "

x26-ii) Outline informed consent procedures

Outline informed consent procedures e.g., if consent was obtained offline or online (how? Checkbox, etc.?), and what information was provided (see 4a-ii). See [6] for some items to be included in informed consent documents.

	1	2	3	4	5	
subitem not at all important	○	○	○	○	◉	essential

Clear selection

Does your paper address subitem X26-ii?

Copy and paste relevant sections from the manuscript (include quotes in quotation marks "like this" to indicate direct quotes from your manuscript), or elaborate on this item by providing additional information not in the ms, or briefly explain why the item is not applicable/relevant for your study

"Participants provide their contact information to the Pitt + Me or BuildClinical systems after accessing the study advertisement. Trained research assistants then contact families and conduct the initial screening to determine study eligibility. If determined eligible, a future call is scheduled to obtain consent from the parent, and assent from the child to participate. After consent, parents are provided a Qualtrics link to complete the initial baseline assessment."

X26-iii) Safety and security procedures

Safety and security procedures, incl. privacy considerations, and any steps taken to reduce the likelihood or detection of harm (e.g., education and training, availability of a hotline)

	1	2	3	4	5	
subitem not at all important	○	○	○	○	◉	essential

Clear selection

Does your paper address subitem X26-iii?

Copy and paste relevant sections from the manuscript (include quotes in quotation marks "like this" to indicate direct quotes from your manuscript), or elaborate on this item by providing additional information not in the ms, or briefly explain why the item is not applicable/relevant for your study

"All study data are deidentified and stored securely."

RESULTS

13a) For each group, the numbers of participants who were randomly assigned, received intended treatment, and were analysed for the primary outcome

NPT: The number of care providers or centers performing the intervention in each group and the number of patients treated by each care provider in each center

Does your paper address CONSORT subitem 13a? *

Copy and paste relevant sections from the manuscript (include quotes in quotation marks "like this" to indicate direct quotes from your manuscript), or elaborate on this item by providing additional information not in the ms, or briefly explain why the item is not applicable/relevant for your study

NA data collection and analyses not complete as the current manuscript describes a protocol.

13b) For each group, losses and exclusions after randomisation, together with reasons

Does your paper address CONSORT subitem 13b? (NOTE: Preferably, this is shown in a CONSORT flow diagram) *

Copy and paste relevant sections from the manuscript (include quotes in quotation marks "like this" to indicate direct quotes from your manuscript), or elaborate on this item by providing additional information not in the ms, or briefly explain why the item is not applicable/relevant for your study

NA data collection and analyses not complete as the current manuscript describes a protocol.

13b-i) Attrition diagram

Strongly recommended: An attrition diagram (e.g., proportion of participants still logging in or using the intervention/comparator in each group plotted over time, similar to a survival curve) or other figures or tables demonstrating usage/dose/engagement.

	1	2	3	4	5	
subitem not at all important	○	○	○	○	◉	essential

Clear selection

Does your paper address subitem 13b-i?

Copy and paste relevant sections from the manuscript or cite the figure number if applicable (include quotes in quotation marks "like this" to indicate direct quotes from your manuscript), or elaborate on this item by providing additional information not in the ms, or briefly explain why the item is not applicable/relevant for your study

NA data collection and analyses not complete as the current manuscript describes a protocol.

14a) Dates defining the periods of recruitment and follow-up

Does your paper address CONSORT subitem 14a? *

Copy and paste relevant sections from the manuscript (include quotes in quotation marks "like this" to indicate direct quotes from your manuscript), or elaborate on this item by providing additional information not in the ms, or briefly explain why the item is not applicable/relevant for your study

NA data collection and analyses not complete as the current manuscript describes a protocol.
"Study recruitment began in December 2022. As of December 2024, we have recruited just over two thirds (n = 224) of our target sampleof 324 parent-child dyads. Of these, 72 parents have been assigned to the standalone condition, 71 to the coach condition, and 71 to the control condition. Recruitment is ongoing and completion is expected in another 12 to 14 months. Follow-up data collection is expected to be completed by the end of 2026."

14a-i) Indicate if critical “secular events” fell into the study period

Indicate if critical “secular events” fell into the study period, e.g., significant changes in Internet resources available or “changes in computer hardware or Internet delivery resources”

	1	2	3	4	5	
subitem not at all important	○	○	○	○	◉	essential

Clear selection

Does your paper address subitem 14a-i?

Copy and paste relevant sections from the manuscript (include quotes in quotation marks "like this" to indicate direct quotes from your manuscript), or elaborate on this item by providing additional information not in the ms, or briefly explain why the item is not applicable/relevant for your study

NA data collection and analyses not complete as the current manuscript describes a protocol.

14b) Why the trial ended or was stopped (early)

Does your paper address CONSORT subitem 14b? *

Copy and paste relevant sections from the manuscript (include quotes in quotation marks "like this" to indicate direct quotes from your manuscript), or elaborate on this item by providing additional information not in the ms, or briefly explain why the item is not applicable/relevant for your study

NA data collection and analyses not complete as the current manuscript describes a protocol.

15) A table showing baseline demographic and clinical characteristics for each group

NPT: When applicable, a description of care providers (case volume, qualification, expertise, etc.) and centers (volume) in each group

Does your paper address CONSORT subitem 15? *

Copy and paste relevant sections from the manuscript (include quotes in quotation marks "like this" to indicate direct quotes from your manuscript), or elaborate on this item by providing additional information not in the ms, or briefly explain why the item is not applicable/relevant for your study

NA data collection and analyses not complete as the current manuscript describes a protocol.

15-i) Report demographics associated with digital divide issues

In ehealth trials it is particularly important to report demographics associated with digital divide issues, such as age, education, gender, social-economic status, computer/Internet/ehealth literacy of the participants, if known.

	1	2	3	4	5	
subitem not at all important	○	○	○	◉	○	essential

Clear selection

Does your paper address subitem 15-i? *

Copy and paste relevant sections from the manuscript (include quotes in quotation marks "like this" to indicate direct quotes from your manuscript), or elaborate on this item by providing additional information not in the ms, or briefly explain why the item is not applicable/relevant for your study

NA data collection and analyses not complete as the current manuscript describes a protocol.

16) For each group, number of participants (denominator) included in each analysis and whether the analysis was by original assigned groups

16-i) Report multiple “denominators” and provide definitions

Report multiple “denominators” and provide definitions: Report N’s (and effect sizes) “across a range of study participation [and use] thresholds” [1], e.g., N exposed, N consented, N used more than x times, N used more than y weeks, N participants “used” the intervention/comparator at specific pre-defined time points of interest (in absolute and relative numbers per group). Always clearly define “use” of the intervention.

	1	2	3	4	5	
subitem not at all important	○	○	○	○	◉	essential

Clear selection

Does your paper address subitem 16-i? *

Copy and paste relevant sections from the manuscript (include quotes in quotation marks "like this" to indicate direct quotes from your manuscript), or elaborate on this item by providing additional information not in the ms, or briefly explain why the item is not applicable/relevant for your study

NA data collection and analyses not complete as the current manuscript describes a protocol.

16-ii) Primary analysis should be intent-to-treat

Primary analysis should be intent-to-treat, secondary analyses could include comparing only “users”, with the appropriate caveats that this is no longer a randomized sample (see 18-i).

	1	2	3	4	5	
subitem not at all important	○	○	○	○	◉	essential

Clear selection

Does your paper address subitem 16-ii?

Copy and paste relevant sections from the manuscript (include quotes in quotation marks "like this" to indicate direct quotes from your manuscript), or elaborate on this item by providing additional information not in the ms, or briefly explain why the item is not applicable/relevant for your study

NA data collection and analyses not complete as the current manuscript describes a protocol.

17a) For each primary and secondary outcome, results for each group, and the estimated effect size and its precision (such as 95% confidence interval)

Does your paper address CONSORT subitem 17a? *

Copy and paste relevant sections from the manuscript (include quotes in quotation marks "like this" to indicate direct quotes from your manuscript), or elaborate on this item by providing additional information not in the ms, or briefly explain why the item is not applicable/relevant for your study

NA data collection and analyses not complete as the current manuscript describes a protocol.

17a-i) Presentation of process outcomes such as metrics of use and intensity of use

In addition to primary/secondary (clinical) outcomes, the presentation of process outcomes such as metrics of use and intensity of use (dose, exposure) and their operational definitions is critical. This does not only refer to metrics of attrition (13-b) (often a binary variable), but also to more continuous exposure metrics such as “average session length”. These must be accompanied by a technical description how a metric like a “session” is defined (e.g., timeout after idle time) [1] (report under item 6a).

	1	2	3	4	5	
subitem not at all important	○	○	○	○	◉	essential

Clear selection

Does your paper address subitem 17a-i?

Copy and paste relevant sections from the manuscript (include quotes in quotation marks "like this" to indicate direct quotes from your manuscript), or elaborate on this item by providing additional information not in the ms, or briefly explain why the item is not applicable/relevant for your study

NA data collection and analyses not complete as the current manuscript describes a protocol.

17b) For binary outcomes, presentation of both absolute and relative effect sizes is recommended

Does your paper address CONSORT subitem 17b? *

Copy and paste relevant sections from the manuscript (include quotes in quotation marks "like this" to indicate direct quotes from your manuscript), or elaborate on this item by providing additional information not in the ms, or briefly explain why the item is not applicable/relevant for your study

NA data collection and analyses not complete as the current manuscript describes a protocol.

18) Results of any other analyses performed, including subgroup analyses and adjusted analyses, distinguishing pre-specified from exploratory

Does your paper address CONSORT subitem 18? *

Copy and paste relevant sections from the manuscript (include quotes in quotation marks "like this" to indicate direct quotes from your manuscript), or elaborate on this item by providing additional information not in the ms, or briefly explain why the item is not applicable/relevant for your study

NA data collection and analyses not complete as the current manuscript describes a protocol.

18-i) Subgroup analysis of comparing only users

A subgroup analysis of comparing only users is not uncommon in ehealth trials, but if done, it must be stressed that this is a self-selected sample and no longer an unbiased sample from a randomized trial (see 16-iii).

	1	2	3	4	5	
subitem not at all important	○	○	○	○	◉	essential

Clear selection

Does your paper address subitem 18-i?

Copy and paste relevant sections from the manuscript (include quotes in quotation marks "like this" to indicate direct quotes from your manuscript), or elaborate on this item by providing additional information not in the ms, or briefly explain why the item is not applicable/relevant for your study

NA data collection and analyses not complete as the current manuscript describes a protocol.

19) All important harms or unintended effects in each group
(for specific guidance see CONSORT for harms)

Does your paper address CONSORT subitem 19? *

Copy and paste relevant sections from the manuscript (include quotes in quotation marks "like this" to indicate direct quotes from your manuscript), or elaborate on this item by providing additional information not in the ms, or briefly explain why the item is not applicable/relevant for your study

NA data collection and analyses not complete as the current manuscript describes a protocol.

19-i) Include privacy breaches, technical problems

Include privacy breaches, technical problems. This does not only include physical "harm" to participants, but also incidents such as perceived or real privacy breaches [1], technical problems, and other unexpected/unintended incidents. "Unintended effects" also includes unintended positive effects [2].

	1	2	3	4	5	
subitem not at all important	○	○	○	○	◉	essential

Clear selection

Does your paper address subitem 19-i?

Copy and paste relevant sections from the manuscript (include quotes in quotation marks "like this" to indicate direct quotes from your manuscript), or elaborate on this item by providing additional information not in the ms, or briefly explain why the item is not applicable/relevant for your study

NA data collection and analyses not complete as the current manuscript describes a protocol.

19-ii) Include qualitative feedback from participants or observations from staff/researchers

Include qualitative feedback from participants or observations from staff/researchers, if available, on strengths and shortcomings of the application, especially if they point to unintended/unexpected effects or uses. This includes (if available) reasons for why people did or did not use the application as intended by the developers.

	1	2	3	4	5	
subitem not at all important	○	○	◉	○	○	essential

Clear selection

Does your paper address subitem 19-ii?

Copy and paste relevant sections from the manuscript (include quotes in quotation marks "like this" to indicate direct quotes from your manuscript), or elaborate on this item by providing additional information not in the ms, or briefly explain why the item is not applicable/relevant for your study

NA data collection and analyses not complete as the current manuscript describes a protocol.

DISCUSSION

22) Interpretation consistent with results, balancing benefits and harms, and considering other relevant evidence

NPT: In addition, take into account the choice of the comparator, lack of or partial blinding, and unequal expertise of care providers or centers in each group

22-i) Restate study questions and summarize the answers suggested by the data, starting with primary outcomes and process outcomes (use)

Restate study questions and summarize the answers suggested by the data, starting with primary outcomes and process outcomes (use).

	1	2	3	4	5	
subitem not at all important	○	○	○	○	◉	essential

Clear selection

Does your paper address subitem 22-i? *

Copy and paste relevant sections from the manuscript (include quotes in quotation marks "like this" to indicate direct quotes from your manuscript), or elaborate on this item by providing additional information not in the ms, or briefly explain why the item is not applicable/relevant for your study

"Mobile health (mHealth) parenting programs have the potential to improve outcomes for parents and children [13]. Further, supportive accountability, defined as a degree of human interaction throughout the program, has the potential to enhance outcomes for mHealth interventions by increasing motivation and engagement [17]. The current study aims to add to the literature base on effective mHealth interventions to treat disruptive behaviors in children. The study will allow us to test the degree to which the UseIt! app can modify parenting (i.e., skill acquisition and utilization) and whether such target engagement is associated with symptom reduction. The UseIt! mHealth system also allows researchers unique access to data on child behaviors and parent skills tracked on a daily basis. This data will not only allow parents to track behaviors and skills for themselves (allowing parents to visualize progress) but will also allow researchers a system to track change over time. The coaching model used in the study is highly scalable, providing a flexible model of coaching that can be tailored to a variety of clinical conditions and service settings.We expect that those assigned to the coach condition will have the largest increases in parent knowledge and skill use, along with the largest decreases in child behavior problems. We also expect that those assigned to the UseIt! standalone app condition will report greater improvements in parent knowledge, skill use, and child behavioral concerns, compared to the control app condition."

Data collection and analyses not complete.

22-ii) Highlight unanswered new questions, suggest future research

Highlight unanswered new questions, suggest future research.

	1	2	3	4	5	
subitem not at all important	○	○	○	○	◉	essential

Clear selection

Does your paper address subitem 22-ii?

Copy and paste relevant sections from the manuscript (include quotes in quotation marks "like this" to indicate direct quotes from your manuscript), or elaborate on this item by providing additional information not in the ms, or briefly explain why the item is not applicable/relevant for your study

"In summary, the current study aims to address a gap in the literature regarding the feasibility, effectiveness, and utility of a smartphone-based application that includes a coach-assisted arm for treating disruptive behaviors in young children. Digital therapeutics have the potential to enhance the reach and scalability of skills-based psychosocial interventions, as even small effects can be meaningful on a population level if the intervention can be delivered efficiently on a large scale at a low cost. The UseIt! mHealth system is able to deliver therapeutic content to parents across a variety of settings and has the potential for meaningful impact. Findings from the current trial will advance scientific knowledge and have the potential to enhance clinical practice."

Data collection and analyses not complete.

20) Trial limitations, addressing sources of potential bias, imprecision, and, if relevant, multiplicity of analyses

20-i) Typical limitations in ehealth trials

Typical limitations in ehealth trials: Participants in ehealth trials are rarely blinded. Ehealth trials often look at a multiplicity of outcomes, increasing risk for a Type I error. Discuss biases due to non-use of the intervention/usability issues, biases through informed consent procedures, unexpected events.

	1	2	3	4	5	
subitem not at all important	○	○	○	○	◉	essential

Clear selection

Does your paper address subitem 20-i? *

Copy and paste relevant sections from the manuscript (include quotes in quotation marks "like this" to indicate direct quotes from your manuscript), or elaborate on this item by providing additional information not in the ms, or briefly explain why the item is not applicable/relevant for your study

"A few limitations are also worth noting. First, we expect up to ten percent attrition over the course of the study. We have taken several steps to minimize attrition, including increasing participant compensation at each time point. We also factored 10% attrition into our power analysis to determine sample size, ensuring that the trial will still be fully powered. Another limitation is that outcome measures are based primarily on parent report. This limitation is mitigated by substantial evidence that parents are accurate reporters of child behavior problems."

21) Generalisability (external validity, applicability) of the trial findings

NPT: External validity of the trial findings according to the intervention, comparators, patients, and care providers or centers involved in the trial

21-i) Generalizability to other populations

Generalizability to other populations: In particular, discuss generalizability to a general Internet population, outside of a RCT setting, and general patient population, including applicability of the study results for other organizations

	1	2	3	4	5	
subitem not at all important	○	○	○	○	◉	essential

Clear selection

Does your paper address subitem 21-i?

Copy and paste relevant sections from the manuscript (include quotes in quotation marks "like this" to indicate direct quotes from your manuscript), or elaborate on this item by providing additional information not in the ms, or briefly explain why the item is not applicable/relevant for your study

Analyses not complete as the current manuscript describes a protocol of a forthcoming RCT.

21-ii) Discuss if there were elements in the RCT that would be different in a routine application setting

Discuss if there were elements in the RCT that would be different in a routine application setting (e.g., prompts/reminders, more human involvement, training sessions or other co-interventions) and what impact the omission of these elements could have on use, adoption, or outcomes if the intervention is applied outside of a RCT setting.

	1	2	3	4	5	
subitem not at all important	○	○	○	○	●	essential

Clear selection

Does your paper address subitem 21-ii?

Copy and paste relevant sections from the manuscript (include quotes in quotation marks "like this" to indicate direct quotes from your manuscript), or elaborate on this item by providing additional information not in the ms, or briefly explain why the item is not applicable/relevant for your study

Analyses not complete as the current manuscript describes a protocol of a forthcoming RCT.

OTHER INFORMATION

23) Registration number and name of trial registry

Does your paper address CONSORT subitem 23? *

Copy and paste relevant sections from the manuscript (include quotes in quotation marks "like this" to indicate direct quotes from your manuscript), or elaborate on this item by providing additional information not in the ms, or briefly explain why the item is not applicable/relevant for your study

Trial Registration: ClinicalTrials.gov registration number: NCT05647772.

24) Where the full trial protocol can be accessed, if available

Does your paper address CONSORT subitem 24? *

Cite a Multimedia Appendix, other reference, or copy and paste relevant sections from the manuscript (include quotes in quotation marks "like this" to indicate direct quotes from your manuscript), or elaborate on this item by providing additional information not in the ms, or briefly explain why the item is not applicable/relevant for your study

Will include in future publication, the current study is a protocol.

25) Sources of funding and other support (such as supply of drugs), role of funders

Does your paper address CONSORT subitem 25? *

Copy and paste relevant sections from the manuscript (include quotes in quotation marks "like this" to indicate direct quotes from your manuscript), or elaborate on this item by providing additional information not in the ms, or briefly explain why the item is not applicable/relevant for your study

"This study was supported by grants to the first author from NIH (R01HD106930; R34MH106619; R21HD090145; K01MH093508) and the Klingenstein Third Generation Foundation (Fellowship in Access to Care)."

X27) Conflicts of Interest (not a CONSORT item)

X27-i) State the relation of the study team towards the system being evaluated

In addition to the usual declaration of interests (financial or otherwise), also state the relation of the study team towards the system being evaluated, i.e., state if the authors/evaluators are distinct from or identical with the developers/sponsors of the intervention.

	1	2	3	4	5	
subitem not at all important	○	○	○	○	◉	essential

Clear selection

Does your paper address subitem X27-i?

Copy and paste relevant sections from the manuscript (include quotes in quotation marks "like this" to indicate direct quotes from your manuscript), or elaborate on this item by providing additional information not in the ms, or briefly explain why the item is not applicable/relevant for your study

This study was supported by grants to the first author from NIH (R01HD106930; R34MH106619; R21HD090145; K01MH093508) and the Klingenstein Third Generation Foundation (Fellowship in Access to Care).

About the CONSORT EHEALTH checklist

As a result of using this checklist, did you make changes in your manuscript? *

- ○ yes, major changes
- ◉ yes, minor changes
- ○ no

What were the most important changes you made as a result of using this checklist?

Clarification of the methods section.

How much time did you spend on going through the checklist INCLUDING making changes in your manuscript *

Two total hours including making changes

As a result of using this checklist, do you think your manuscript has improved? *

- (x) yes
- ( ) no
- ( ) Other:

Would you like to become involved in the CONSORT EHEALTH group?

This would involve for example becoming involved in participating in a workshop and writing an "Explanation and Elaboration" document

- ( ) yes
- (x) no
- ( ) Other:

Clear selection

Any other comments or questions on CONSORT EHEALTH

Your answer

STOP - Save this form as PDF before you click submit

To generate a record that you filled in this form, we recommend to generate a PDF of this page (on a Mac, simply select "print" and then select "print as PDF") before you submit it.

When you submit your (revised) paper to JMIR, please upload the PDF as supplementary file.

Don't worry if some text in the textboxes is cut off, as we still have the complete information in our database. Thank you!

Final step: Click submit !

Click submit so we have your answers in our database!

Submit Clear form

Never submit passwords through Google Forms.

This content is neither created nor endorsed by Google. - Terms of Service - Privacy Policy

Does this form look suspicious? Report

Google Forms
